# Application of Texture Analysis to Study Small Vessel Disease and Blood–Brain Barrier Integrity

**DOI:** 10.3389/fneur.2017.00327

**Published:** 2017-07-19

**Authors:** Maria del C. Valdés Hernández, Victor González-Castro, Francesca M. Chappell, Eleni Sakka, Stephen Makin, Paul A. Armitage, William H. Nailon, Joanna M. Wardlaw

**Affiliations:** ^1^Department of Neuroimaging Sciences, Centre for Clinical Brain Sciences, University of Edinburgh, Edinburgh, United Kingdom; ^2^Department of Infection, Immunity and Cardiovascular Disease, University of Sheffield, Sheffield, United Kingdom; ^3^Department of Oncology Physics, University of Edinburgh, Edinburgh, United Kingdom

**Keywords:** texture analysis, cerebral small vessel disease, blood–brain barrier, hypertension, age, stroke, leukoaraiosis, perivascular spaces

## Abstract

**Objectives:**

We evaluate the alternative use of texture analysis for evaluating the role of blood–brain barrier (BBB) in small vessel disease (SVD).

**Methods:**

We used brain magnetic resonance imaging from 204 stroke patients, acquired before and 20 min after intravenous gadolinium administration. We segmented tissues, white matter hyperintensities (WMH) and applied validated visual scores. We measured textural features in all tissues pre- and post-contrast and used ANCOVA to evaluate the effect of SVD indicators on the pre-/post-contrast change, Kruskal–Wallis for significance between patient groups and linear mixed models for pre-/post-contrast variations in cerebrospinal fluid (CSF) with Fazekas scores.

**Results:**

Textural “homogeneity” increase in normal tissues with higher presence of SVD indicators was consistently more overt than in abnormal tissues. Textural “homogeneity” increased with age, basal ganglia perivascular spaces scores (*p* < 0.01) and SVD scores (*p* < 0.05) and was significantly higher in hypertensive patients (*p* < 0.002) and lacunar stroke (*p* = 0.04). Hypertension (74% patients), WMH load (median = 1.5 ± 1.6% of intracranial volume), and age (mean = 65.6 years, SD = 11.3) predicted the pre/post-contrast change in normal white matter, WMH, and index stroke lesion. CSF signal increased with increasing SVD post-contrast.

**Conclusion:**

A consistent general pattern of increasing textural “homogeneity” with increasing SVD and post-contrast change in CSF with increasing WMH suggest that texture analysis may be useful for the study of BBB integrity.

## Key Points

- Texture analysis is useful to study small vessel disease (SVD) and blood–brain barrier.
- Results of texture analysis consistent with pathophysiology of SVD.
- Post-contrast texture change indicates more gadolinium in cerebrospinal fluid with increasing disease burden.
- Increasing textural homogeneity with disease severity confirms disease progression pattern.

## Introduction

Small vessel disease (SVD) describes “a syndrome of clinical, cognitive, neuroimaging, and neuropathological findings thought to arise from disease affecting the perforating cerebral arterioles, capillaries, and venules, and the resulting brain damage in the cerebral white and deep gray matter (DGM).” SVD is a common cause of dementia and causes about a fifth of all strokes worldwide (1). Although the cause of cerebral SVD is unknown, increasing evidence indicates that the microvessel endothelium, i.e., the blood–brain barrier (BBB), plays a key role in SVD pathogenesis (2–6).

Blood–brain barrier functional integrity is commonly studied through the analysis of quantitative data obtained using dynamic contrast-enhanced (DCE) magnetic resonance imaging (MRI) (7). Various approaches to assess BBB integrity exist, ranging from plotting the average signal enhancement in a tissue or region over time and calculating the area under the curve (8) to modeling the tracer kinetics in tissue assuming different conditions (7). However, scanner noise, drift, and intrinsic tissue properties affect the average signal enhancement and need to be accounted for, and lack of a valid method to determine the microvessel surface area limits current ability to estimate actual permeability (8, 9). Hence, we evaluate an alternative approach to document contrast leakage across the BBB through the analysis of some descriptors of cerebrospinal fluid (CSF) and tissues in fluid-attenuation inversion recovery (FLAIR) images before and after gadolinium-based intravenous contrast administration on individuals with SVD. FLAIR is sensitive to even small amounts of this contrast agent. If gadolinium manifests as high signal in CSF on FLAIR when injected intravenously, it can only have reached the CSF by crossing the BBB (10, 11). We hypothesized that the spatial statistical distribution of the signal intensity in a tissue type might provide useful information on tissue changes with changing degree of SVD and could reflect the BBB status including differences pre-/post-contrast injection.

### Rationale and Background of Our Alternative Approach

**Figure 1:**
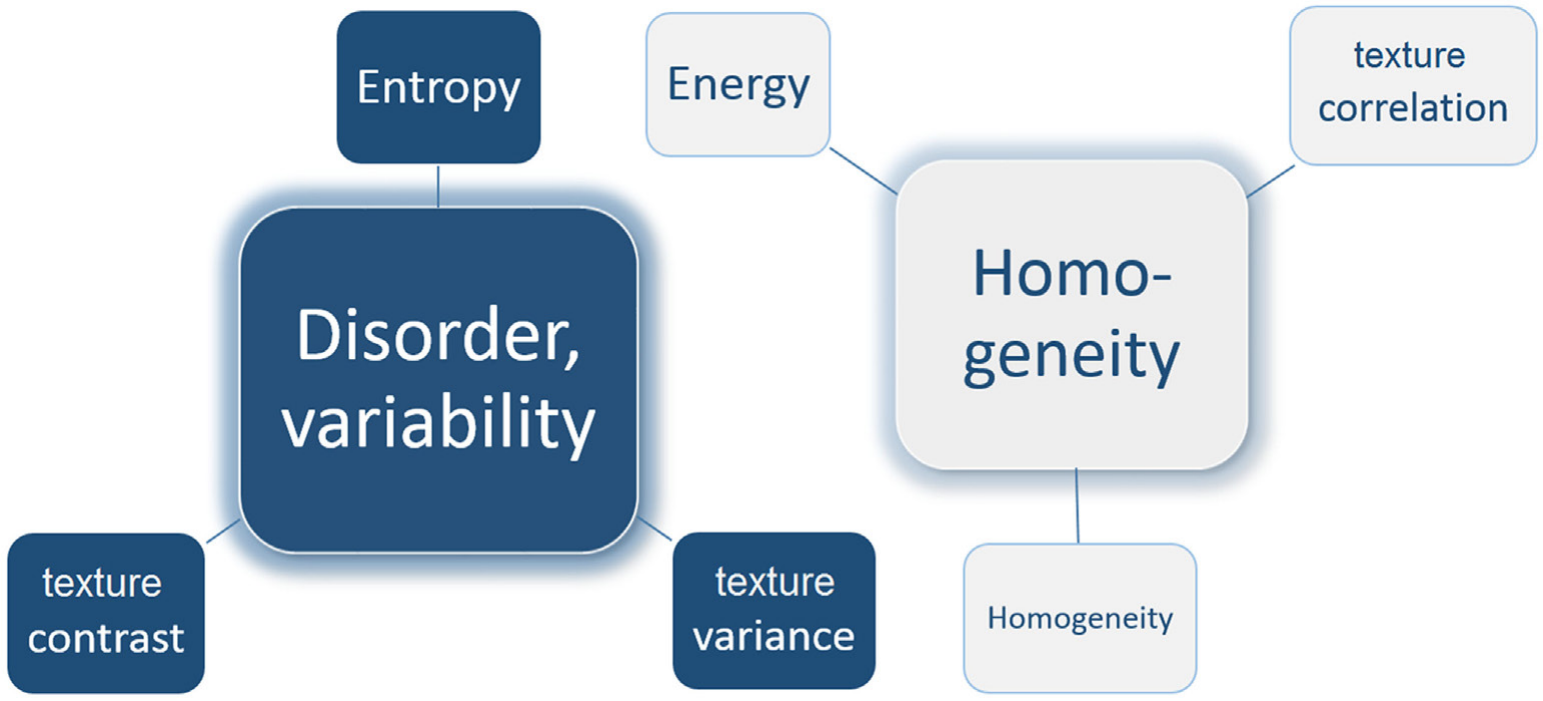
Schematic representation of two groups of texture descriptors. Texture contrast, variance, and entropy express the disorder and variability in the tissue intensities. On the contrary, texture correlation, homogeneity, and energy express the homogeneity of the intensities in the tissue.

The property concerned with the spatial statistical distribution of image intensity levels is the “texture.” Image texture descriptors express, in different ways, the properties of the “patterns” of an image or a section of it: the perceived “lightness,” “uniformity,” “spatial density,” “roughness” or “coarseness,” “regularity,” “linearity,” “directionality,” “randomness,” “fineness,” “smoothness,” “granulation,” etc. There are some intuitive expectations for properties being represented by some descriptors. For example, one might expect the *entropy* descriptor to be higher if the appearance of the gray levels in a region on the image is “coarse” or “rough” and with irregular or non-recurrent patterns, rather than “smooth” or “uniform,” as *entropy* expresses randomness or “disorder.” There are other texture descriptors that, to a different degree, have similar tendency as *entropy*, like those that express the contrast or the variability of the gray levels in the region. These texture descriptors can be grouped to express the heterogeneity, or variability of the intensity levels of a region in an image, as opposed to others that represent homogeneity (Figure 1).

Texture descriptors have been used as input parameters in machine-learning approaches for human tissue classification (i.e., discriminating malignant vs. benign types of tumors) (12–23), for predicting response to cancer treatment (24–27), and for characterizing tumors (13, 28, 29). However, to the best of our knowledge, only one study on brain MRI from our group (30) has analyzed texture descriptors to investigate the properties of apparently normal tissues on routine structural MRI on patients with SVD. It concluded that local signal intensity variations in normal tissues in patients with SVD could be associated with SVD markers, but lacked statistical power due to small sample size (i.e., 42 patients) and did not use contrast-enhanced MRI. We selected six texture descriptors out of the 14 formulated by Haralick et al. (31). These have been used in supervised and semi-supervised machine-learning schemes to analyze DCE-MRI as an alternative to pharmacokinetic models or area under the signal enhancement curve in an attempt to address the low signal specificity (12, 16, 22, 32) and high variability in results (12, 14, 22) (see Data Sheet S1 in Supplementary Material^1^).

### Scientific Questions and Expected Results

In the present study, we investigated, on a population known to have a wide range of SVD severity indicators, (1) whether a manifestation indicative of possible BBB leakage can be detected by texture analysis of FLAIR images comparing pre- vs. post-intravenous contrast agent and (2) whether the level of “heterogeneity” differs in CSF, normal, and abnormal brain tissues for patients with more vs. less clinically and imaging evident SVD. If texture analysis is useful for the study of subtle BBB leakage, given that high SVD scores are correlated with increased BBB leakage (6, 33), we anticipate that: (A) the textural change post-contrast (i.e., after injecting the contrast agent) vs. pre-contrast (i.e., before injecting the intravenous contrast agent) will be bigger in patients with more SVD; (B) a higher BBB leakage, which is more likely with more SVD markers, will make the texture of the signal in the normal and abnormal tissues smoother (i.e., more “homogeneous” texture) due to an increased BBB leakage uniformly distributed across the tissues; (C) indicators of textural “homogeneity” will be of higher values in tissues in patients that had a lacunar stroke rather than in those who had a cortical stroke (34), this being more evident post-contrast, as lacunar strokes provide a useful alert to the presence of SVD; and (D) the same effect (i.e., increased textural homogeneity in tissues) would be seen in patients with hypertension (35), with more perivascular spaces (PVS) seen in the basal ganglia (36) and with higher global SVD scores (33). These expected results will be consistent with the increase in the freedom of movement of the tissue water molecules (i.e., mean diffusivity) with age, which would favor an homogeneous appearance in pathophysiologically similar regions as seen in studies using diffusion tensor images (37, 38).

### Evaluations Done

To test our hypotheses, we evaluated (1) pre–post-contrast change in textural descriptors in CSF with severity of white matter disease and (2) textural characteristics of normal and abnormal tissues against indicators of SVD. As part of the latter, we specifically investigate (2.1) the influence of age on textural “heterogeneity” (i.e., expressed by the texture descriptors that express “variability” or “randomness” in the spatial distribution of the gray levels), expecting that textural “heterogeneity” will decrease in normal and abnormal tissues with increasing age as SVD indicators are more evident (33, 39); (2.2) the comparative values of a selection of the texture descriptors proposed by Haralick and colleagues in normal-appearing white matter (NAWM), DGM, CSF, and abnormal regions [i.e., index infarcts and white matter hyperintensities (WMH)] in patients with: (a) hypertension vs. normotension, (b) cortical vs. lacunar ischemic strokes, (c) few to many PVS in the basal ganglia region (33), (d) low to high WMH load evaluated using the Fazekas scale (40), and (d) low to high SVD scores (41, 42), pre- and post-contrast.

## Materials and Methods

### Subjects

We used data from a prospective study of patients recruited consecutively (*n* = 204, 81 women) with lacunar (*n* = 93) or mild (i.e., mRS <3) cortical (*n* = 111) ischemic stroke with mean age 65.6 years old (SD 11.3 years) who gave written consent to participate on a study of stroke mechanisms (6, 43) and had the valid brain MRI sequences required for the analysis presented here. Diabetes, hypertension, and other vascular risk factors were not criteria for exclusion. Patients with unstable hypertension or diabetes, other non-vascular neurological disorders, major medical conditions including renal failure, contraindications to MRI, unable to give consent, those who had hemorrhagic stroke or whose symptoms resolved within 24 h (i.e., transient ischemic attack) were excluded. The study was approved by the Lothian Ethics of Medical Research Committee (REC 09/81101/54) and the NHS Lothian R+D Office (2009/W/NEU/14) and conducted according to the principles expressed in the Declaration of Helsinki.

### Brain MRI Acquisition

Brain MRI for assessing BBB leakage was conducted between one and three months after stroke [median 38, interquartile range (IQR) 31–54 days], on a 1.5-T GE Signa LX clinical scanner (General Electric, Milwaukee, WI, USA), equipped with a self-shielding gradient set and manufacturer supplied eight-channel-phased array heal coil. In this study we analyze fluid-attenuated inversion recovery (FLAIR) images, acquired with TE 147 ms, TR 9,002 ms, field of view 240 mm × 240 mm, acquisition matrix 256 × 256, slice thickness 5 mm, 1 mm inter-slice gap, and voxel size 0.94 mm × 0.94 mm × 6.5 mm. This sequence was acquired twice: at the beginning of the imaging session (i.e., pre-contrast) and approximately 21 min after an intravenous bolus injection of 0.1 mmol/kg gadoterate meglumine (Gd-DOTA, Dotarem, Guerbet, France) (i.e., post-contrast) (44). However, for tissue segmentation, diffusion-weighted and structural T1-weighted, T2-weighted and gradient echo, acquired as specified elsewhere (43) were also used.

### Image Processing

Tissue segmentation was performed following the protocol described in Ref. (43). Briefly, binary masks of NAWM, CSF, and WMH were obtained using a multispectral segmentation method (45)^2^ with manual editing to correct errors where necessary. Deep gray matter binary masks were obtained automatically using FSL tools^3^ [SUSAN (46), FIRST (47), and FLIRT (48)] and a relevant template (49) using a pipeline developed in-house followed by manual editing. Binary masks from the stroke lesions were obtained semi-automatically by thresholding on FLAIR using Analyze 12.0™ guided by the diffusion-weighted images and neuroradiological advice followed by manual editing. For tissue segmentation all images were co-registered to the pre-contrast FLAIR sequence using FLIRT.

All binary masks (i.e., from NAWM, DGM, WMH, CSF, and stroke lesions) were applied to the pre-contrast and post-contrast FLAIR images thus obtaining images of pre-/post-contrast FLAIR intensities in each tissue type for each patient. Inter-rater reliability on the generation of these masks was: mean dissimilarity index 0.3 and mean volumetric differences on the range of 1.4% (SD 19.7%) to 4.4% (SD 18.7%) of the volumes measured (43). To avoid possible effects of random noise and non-uniformities due to different intensity ranges, intensities were normalized and quantized (i.e., grouped) into 8, 16, 32, and 64 equally spaced “bins” as a trade-off between the number of gray levels and the computational cost of the calculation of the texture descriptors.

For each pre-/post-contrast FLAIR tissue/lesion image, we computed the texture descriptors of six second order statistics that intuitively expressed either “variability” or “homogeneity” of the texture in the imaged tissue, selected from the 14 texture descriptors proposed by Haralick et al. (31); giving, therefore, six individual texture descriptors for each tissue type twice. They were all extracted from the gray level co-occurrence matrix (GLCM), which is a matrix reflecting the distribution of the relationships between pairs of pixels in a quantized 2D image (Figure 1; Figure S1 in Supplementary Material).

As images were quantized in 8, 16, 32, and 64 gray levels, we obtained GLCM matrices of 8 × 8, 16 × 16, 32 × 32, and 64 × 64 sizes. The selected texture descriptors were as follows: *GLCM contrast* (a measure of intensity variations between a reference pixel and its neighbor), sum of squares (referred to as *GLCM variance*, a measure of the dispersion of the values around the mean of combinations of reference and neighboring pixels), *entropy* (an expression of the chaos or randomness in the texture), *GLCM correlation* (a measure of the linear dependency of intensities in the GLCM), *homogeneity* (measures the local homogeneity of the texture), and *energy* (measures the uniformity of a texture).

For each axial slice in a volume, four GLCMs of the same dimension (i.e., either 8 × 8 or 16 × 16 or 32 × 32 or 64 × 64) were calculated, each one with distance *d* = 1 and a different orientation θ (0°, 45°, 90°, and 135°). For each orientation, we summed the GLCMs from all slices to obtain a global GLCM for each orientation θ on the whole volume. Subsequently, we normalized these four global GLCMs so that each one expressed a joint probability distribution of the co-occurrences of the voxels in a certain orientation θ and distance *d*. The texture descriptors previously mentioned were calculated from each of the four “global” GLCMs. The final value of the texture descriptor on the FLAIR image of a tissue type was obtained by averaging its components in the four directions. This last step provided some invariance to rotation.

### Statistical Analyses

To evaluate tendency in the pre-/post-contrast changes in CSF with severity of white matter disease, a linear mixed model was fitted for each texture variable. This accounted for there being two (pre- and post-contrast) measurements per patient. Model fit was checked by examining residual plots and collinearity by variance inflation factors and condition indices. Predictors were chosen on grounds of clinical plausibility, namely, index stroke subtype, age, PVS score in the basal ganglia and centrum semiovale, Fazekas score, mean arterial pressure, and diagnosis of diabetes, with a binary predictor to indicate whether the outcome variable had been measured pre- or post-contrast. We then added an interaction term to each model to see if the difference between pre- and post-contrast measurements varied with Fazekas scores. We used PROC MIXED in SAS 9.4^4^.

To evaluate the influence of the intravenous contrast agent on the distributions of the intensities in normal and abnormal tissues, we analyzed the pre-/post-contrast difference in the variance-to-mean ratio (DVMR, equals to VMR post-contrast minus VMR pre-contrast) on the FLAIR signal intensities [see equation (9) in Supplementary Material]. To evaluate the effect of age, hypertension, WMH load and basal ganglia PVS scores on the pre-/post-intravenous contrast differences (50), we used ANCOVA.

To evaluate the textural characteristics of normal and abnormal tissues against indicators of SVD, we used the Kruskal–Wallis test in IBM SPSS Statistics ver. 21 testing the null hypothesis that the distribution of the values of the six texture descriptors selected in the tissues/lesions (i.e., NAWM, WMH, etc.) was the same across all patient categories (i.e., hypertensive vs. normotensive, lacunar vs. cortical, SVD scores, etc.). The Mood’s Median test, also in IBM SPSS Statistics ver. 21, was used to evaluate the null hypothesis that the medians of the texture descriptors in the tissues were the same across different categories (i.e., hypertensive vs. normotensive, lacunar vs. cortical stroke, different basal ganglia PVS scores and different SVD scores).

Of note, although we are presenting the results obtained from GLCMs of *N* = 16 (i.e., the gray levels of the intensity normalized FLAIR images were reduced to 16 after applying uniform quantization), different quantization levels (i.e., *N* = 8, *N* = 32, and *N* = 64) yielded almost identical results and are available upon request.

Finally, to validate our analyses, we evaluated the interhemispheric differences (i.e. ipsilateral vs. contralateral) within-patient in the entropy values on regions of interest (ROIs) manually placed on one slice of the NAWM (43) in a subsample of 39 patients randomly selected using Wilcoxon’s test, and the differences between the ROIs using the Friedman’s test.

## Results

### Sample Characteristics

The sample clinical characteristics have been published previously (44). Those included in our analyses, which are relevant to this study are summarized in Table 1.

**Table 1:** Clinical and imaging variables from the sample relevant for this study.

Clinical/imaging variable	No. of patients (% with respect to the total)
**Hypertension**	
Hypertensive	151 (74%)
Normotensive	53 (26%)
**Index stroke type**	
Cortical	111 (54.4%)
Lacunar	93 (45.6%)
**Fazekas white matter hyperintensities scores**	
0	7 (3.43%)
1	16 (7.84%)
2	76 (37.26%)
3	23 (11.27%)
4	30 (14.71%)
5	20 (9.80%)
6	32 (15.69%)
**Basal ganglia perivascular spaces score**	
0	4 (2%)
1	103 (50.5%)
2	55 (27%)
3	26 (12.7%)
4	16 (7.8%)
**Small vessel disease score**	
0	69 (33.8%)
1	49 (24%)
2	47 (23%)
3	26 (12.7%)
4	13 (6.4%)

### Texture before (i.e., Pre-) vs. after (i.e., Post-) the Intravenous Contrast Injection

#### Post-Contrast Changes in Texture

**Figure 2:**
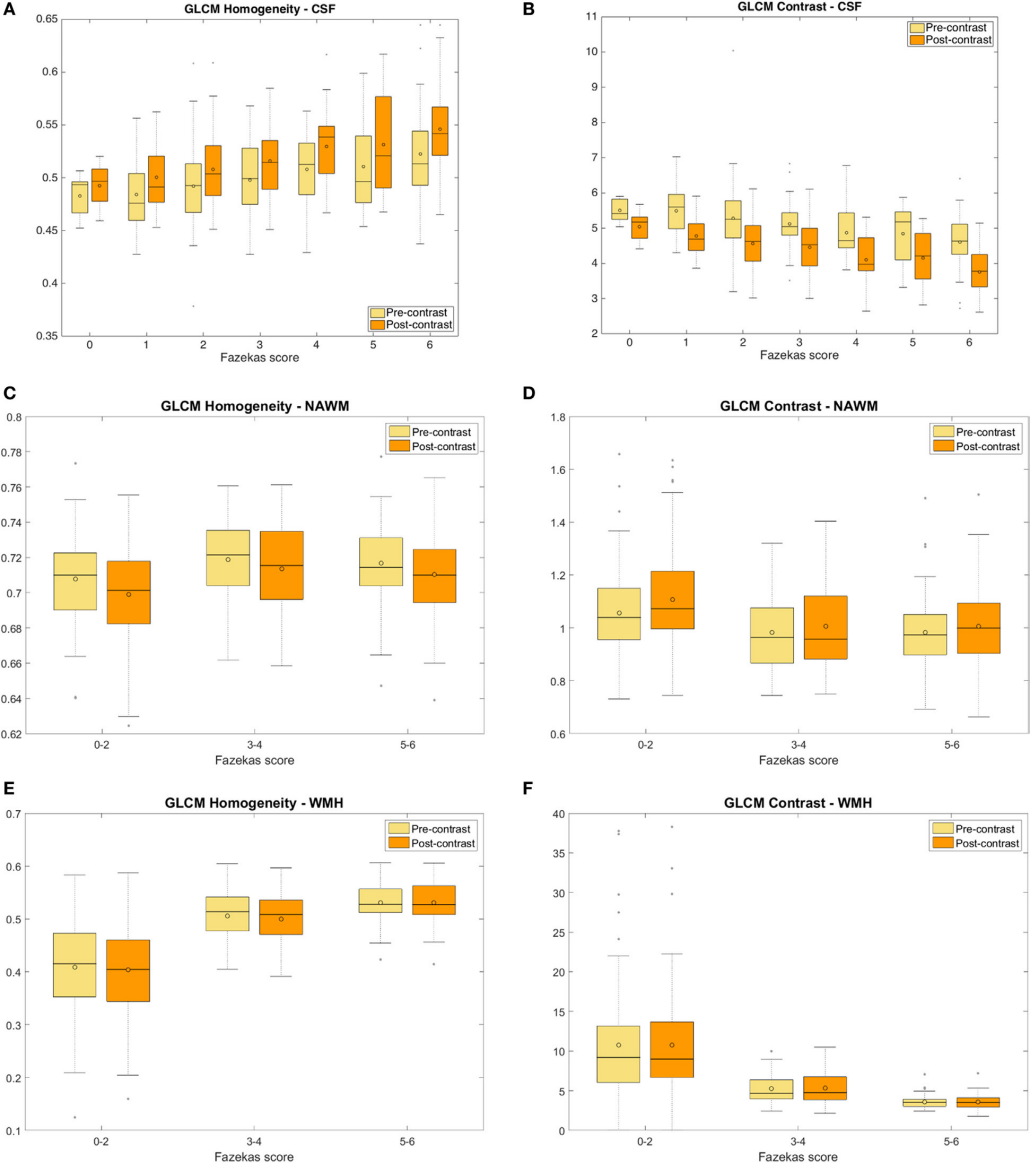
Measures of the pre- and post-contrast FLAIR homogeneity **(A)** and gray level co-occurrence matrix (GLCM) contrast **(B)** of the cerebrospinal fluid (CSF) for different total Fazekas scores showing the variation in CSF texture with increasing white matter hyperintensities (WMH) burden; and measures of pre- and post-contrast FLAIR GLCM homogeneity **(C,E)** and GLCM contrast **(D,F)** of the normal-appearing white matter (NAWM) **(C,D)** and WMH **(E,F)** for different Fazekas score groups.

All texture descriptors experienced a post-contrast change with respect to their pre-contrast values (Table S2.1 in Supplementary Material). The pre-/post-contrast differences were small, and there was no distinct pattern of pre-/post-contrast increase/decrease “homogeneity” or “variability” in most tissues, except for CSF. The differences between pre- and post-contrast CSF texture (median and IQR values in the Table S2.1 in Supplementary Material) increased with worsening WMH (i.e., the Fazekas score) (Figures 2A,B). The trajectory post-contrast is steeper with increasing WMH burden, indicating that more gadolinium enters into the CSF with worsening white matter disease. Table 2 shows the results of the linear mixed model for each textural feature extracted from the CSF on the pre- and post-contrast FLAIR images. The difference between pre- and post-contrast CSF measurements was always significant after adjustment for Fazekas scores, age, stroke subtype, PVS in the basal ganglia and centrum semiovale, mean arterial pressure, and diabetes for all outcome variables, tests of interaction between Fazekas scores and the pre- vs. post-contrast changes showed a significant interaction confirming the increased leakage of gadolinium into CSF with increasing WMH (Table 2). Residual plots suggested that the model fit varied from adequate-to-good for each outcome variable. Collinearity diagnostics were satisfactory, with all variance inflation factors under 2 and condition indices below 8.

**Table 2:** Pre- vs. post-contrast in cerebrospinal fluid and Fazekas scores adjusted for each other and baseline stroke subtype, age, perivascular spaces in basal ganglia and centrum semiovale, mean arterial pressure, and baseline diagnosis of diabetes.

Outcome	Effect	Estimate		Interaction	
		*B* [95% confidence interval (CI)]	*p*-Value	Pre vs. post × Fazekas	*p*-Value
Gray level co-occurrence matrix (GLCM) variance	Pre_vs_Post	−1.069 (−1.173, −0.966)	**<0.0001**	−0.0612 (−0.121, −0.00107)	**0.046**
	Fazekas scores	−0.0319 (−0.126, 0.0620)	0.50		
GLCM contrast	Pre_vs_Post	−0.729 (−0.794, −0.665)	**<0.0001**	−0.0294 (−0.0669, 0.00811)	0.12
	Fazekas scores	−0.109 (−0.182, −0.0353)	**0.0039**		
GLCM correlation	Pre_vs_Post	0.0111 (0.00888, 0.0132)	**<0.0001**	0.000821 (−0.000450, 0.00209)	0.20
	Fazekas scores	0.00555 (0.00195, 0.00914)	**0.0026**		
Energy	Pre_vs_Post	0.00142 (0.00111, 0.00173)	**<0.0001**	0.000190 (0.0000120, 0.000368)	**0.037**
	Fazekas scores	0.00101 (0.000332, 0.00170)	**0.0038**		
Entropy	Pre_vs_Post	−0.140 (−0.156, −0.123)	**<0.0001**	−0.0117 (−0.0213, −0.00211)	**0.017**
	Fazekas scores	−0.0329 (−0.0576, −0.00813)	**0.0095**		
Homogeneity	Pre_vs_Post	0.0186 (0.0166, 0.0206)	**<0.0001**	0.00189 (0.000742, 0.00303)	**0.0013**
	Fazekas scores	0.00529 (0.00171, 0.00887)	**0.0040**		

*Results are given as values (95% CI)*.

*p < 0.05 and p < 0.001*.

**Figure 3:**
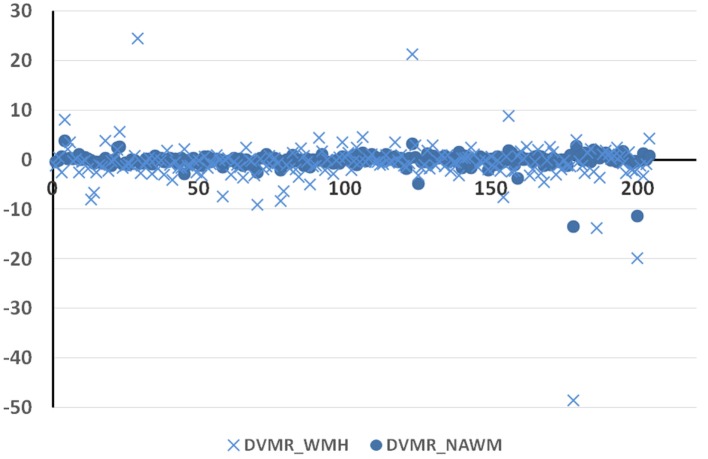
Difference in the variance-to-mean ratio in normal [normal-appearing white matter (NAWM)] and abnormal [white matter hyperintensities (WMH)] tissue between post-contrast and pre-contrast FLAIR images (*y*-axis) on the 204 datasets (*x*-axis).

The intravenous contrast seemed to affect the texture in the abnormal tissues more than in the normal tissues. This is shown by the higher dispersion of the pre-/post-contrast signal intensity differences in variance-to-mean ratios in the WMH (abnormal tissue) with respect to those in the NAWM (normal tissue) (Figure 3).

#### Predictors of Post-Contrast Change in Texture

The FLAIR post-contrast texture descriptors evaluated were strongly and significantly dependent on the pre-contrast texture in all tissue types and CSF, independent of the size of the GLCM. The pre-/post-contrast change in CSF texture was only predicted by age. The CSF texture after contrast became more “homogeneous” in older patients. Hypertension, Fazekas scores, and age predicted the pre-/post-contrast change captured by some of the texture descriptors in NAWM and WMH. Figures 2C–F show the GLCM homogeneity and GLCM contrast of the NAWM and WMH in pre- and post-contrast FLAIR images grouped by Fazekas scores (specifically 0–2, 3–4, and 5–6). The linear dependency of the intensities in the GLCM matrix (i.e., *GLCM correlation*) computed in the index stroke lesion experienced a post-contrast change partially influenced by age. However, textural differences before and after contrast in DGM, if any, were due to chance or unrelated to any of the vascular risk factors/SVD indicators analyzed or the type of stroke. Table 3 shows the results of the ANalysis of COVAriance for the parameters that, in addition to the pre-contrast signal, predicted the post-contrast signal for each tissue type and CSF for images quantized to *N* = 16 gray levels. Similar results were obtained for images quantized to 8, 32, and 64 levels.

**Table 3:** Results of the ANCOVA analysis of the pre-/post-contrast change as per the following model: texture after contrast = *X*_1_ × texture before contrast + *X*_2_ × Age + *X*_3_ × Fazekas scores + *X*_4_ × PVS scores in basal ganglia + *X*_5_ × Hypertension.

Textural descriptor		Predictor	Cerebrospinal fluid	Normal tissues		Abnormal tissues	
				Normal-appearing white matter	Deep Gray Matter	Index Stroke lesion	White matter hyperintensities
Homogeneity	Gray level co-occurrence matrix (GLCM) correlation	**Age**	−1.72 × 10^−6^; 0.99	−2.36 × 10^−4^; 0.087	−1.30 × 10^−4^; 0.38	**9.84 × 10**^−^^4^; 0.038*	1.18 × 10^−4^; 0.67
		Fazekas scores	0.0014; 0.40	0.0019; 0.062	4.74 × 10^−4^; 0.68	6.09 × 10^−4^; 0.86	0.0037; 0.12
		BG-PVS scores	−0.0024; 0.37	−7.81 × 10^−4^; 0.64	3.47 × 10^−4^; 0.85	−0.0074; 0.20	0.0049; 0.14
		**Hypertension**	−0.0023; 0.63	0.0091; 0.0038*	0.0027; 0.41	−0.0036; 0.73	0.0088; 0.16

	Homogeneity	Age	6.56 × 10^−6^; 0.99	−1.20 × 10^−4^; 0.30	−1.28 × 10^−4^; 0.44	1.64 × 10^−4^; 0.51	2.55 × 10^−4^; 0.10
		**Fazekas scores**	0.0015; 0.63	0.0011; 0.20	−0.0016; 0.21	−8.36 × 10^−5^; 0.96	0.0037; 0.0042*
		BG-PVS scores	−0.0061; 0.22	4.52 × 10^−5^; 0.97	0.0013; 0.52	−3.11 × 10^−4^; 0.92	1.057 × 10^−5^; 0.99
		Hypertension	−0.0027; 0.77	0.0045; 0.091	3.68 × 10^−4^; 0.92	0.010; 0.068	0.0053; 0.13

	Energy	Age	−5.57 × 10^−4^; 0.36	1.44 × 10^−5^; 0.67	−1.33 × 10^−5^; 0.71	−2.15 × 10^−5^; 0.54	2.57 × 10^−5^; 0.22
		Fazekas scores	−6.80 × 10^−4^; 0.88	2.22 × 10^−4^; 0.39	−2.78 × 10^−4^; 0.30	−2.32 × 10^−4^; 0.37	−5.98 × 10^−5^; 0.71
		BG-PVS scores	−0.0079; 0.29	2.39 × 10^−4^; 0.57	2.33 × 10^−4^; 0.60	3.63 × 10^−4^; 0.42	1.04 × 10^−4^; 0.69
		Hypertension	−0.0021; 0.88	−4.30 × 10^−4^; 0.58	−3.04 × 10^−4^; 0.71	5.42 × 10^−4^; 0.52	3.28 × 10^−4^; 0.49

Variability	GLCM contrast	**Age**	**−0.0095; 0.058^a^**	3.99 × 10^−4^; 0.57	1.71 × 10^−4^; 0.92	−0.022; 0.077	**−0.017; 0.057^a^**
		**Fazekas scores**	−0.052; 0.14	**−0.010; 0.054^a^**	0.0057; 0.68	0.016; 0.86	−0.16; 0.036*
		BG-PVS scores	0.084; 0.14	0.0035; 0.69	−0.022; 0.31	0.064; 0.68	0.029; 0.79
		**Hypertension**	0.089; 0.40	−0.029; 0.072	0.027; 0.50	−0.21; 0.46	−0.41; 0.048*

	GLCM variance	**Age**	−0.015; 0.016*	−0.0096; 0.042*	−0.0097; 0.24	0.0065; 0.74	−0.019; 0.042*
		**Fazekas scores**	−0.057; 0.22	0.021; 0.55	0.038; 0.54	−0.0080; 0.95	−0.18; 0.013*
		BG-PVS scores	0.114; 0.13	−0.036; 0.53	−0.13; 0.20	−0.12; 0.62	0.098; 0.40
		Hypertension	0.190; 0.17	0.178; 0.096	0.27; 0.14	−0.39; 0.39	−0.20; 0.36

	Entropy	Age	0.0018; 0.69	−6.54 × 10^−4^; 0.44	9.63 × 10^−5^; 0.93	0.0014; 0.35	−9.68 × 10^−4^; 0.33
		Fazekas scores	−0.0068; 0.84	−0.0062; 0.34	0.0038; 0.64	0.0091; 0.39	0.0010; 0.89
		BG-PVS scores	0.068; 0.21	−0.0038; 0.72	−0.015; 0.24	−0.017; 0.34	8.17 × 10^−4^; 0.95
		Hypertension	0.035; 0.72	0.0069; 0.72	0.015; 0.54	−0.034; 0.32	−0.011; 0.62

*Results are given as [predictor (estimate; *p*-value)]*.

*p < 0.05.

*^a^Borderline significance*.

### Tissues’ Texture and Age

**Figure 4:**
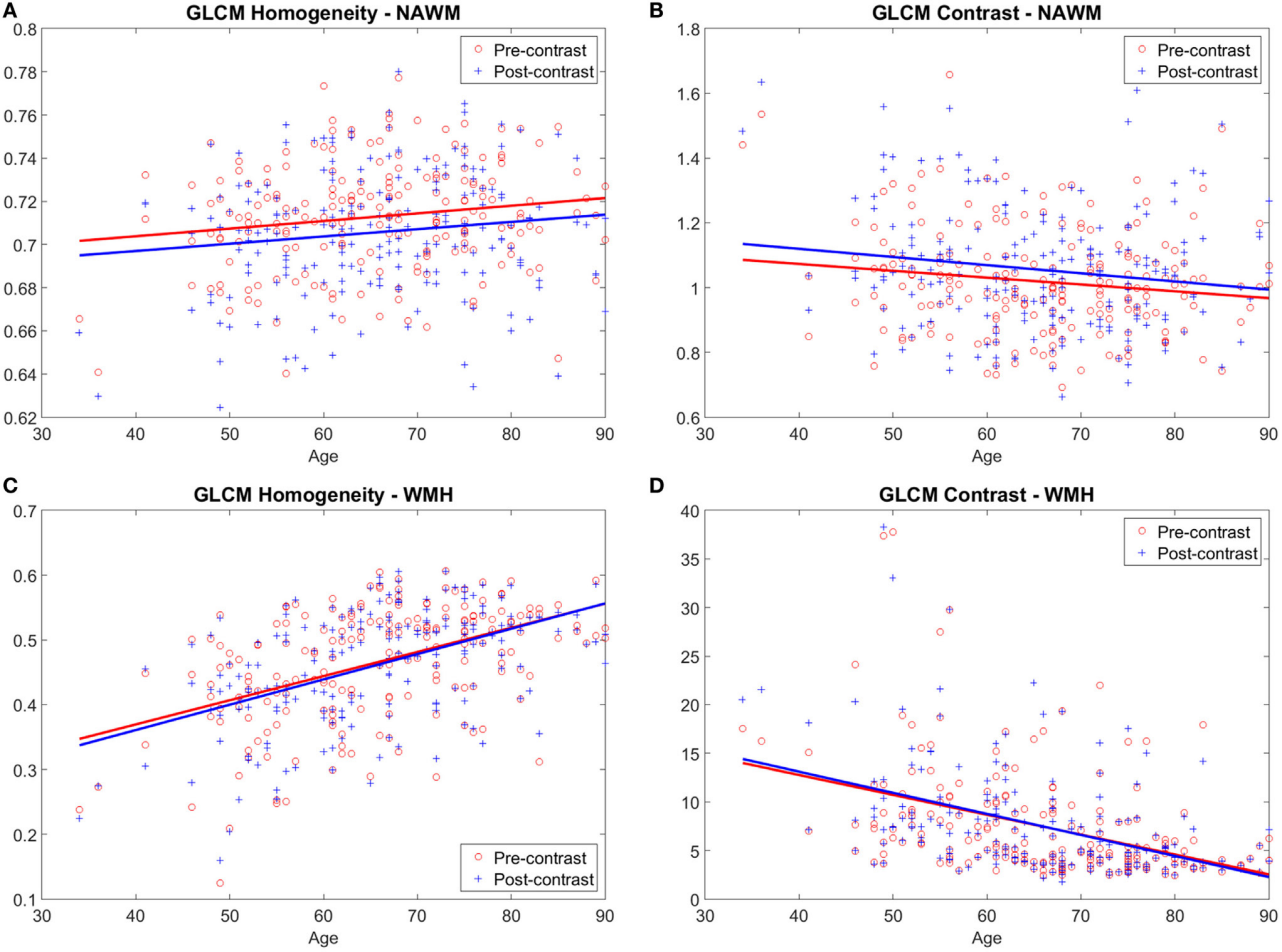
Variation of homogeneity **(A,C)** and variability **(B,D)** of the textures with age. Examples show gray level co-occurrence matrix (GLCM) homogeneity and GLCM contrast in normal-appearing white matter (NAWM) **(A,B)** and white matter hyperintensities (WMH) **(C,D)** obtained from pre- and post-contrast FLAIR images.

All texture descriptors in normal and abnormal tissues varied with age (both in pre- and in post-contrast). Those that expressed the “homogeneity” in texture increased with increasing age, whereas those that expressed variability decreased with increasing age. This tendency was more accentuated in abnormal tissues than in normal tissues (Figure 4; Figures S2.2 and S2.3 in Supplementary Material).

### Tissues’ Texture in Patient Groups

Medians and distributions of all texture descriptors differed significantly between all subgroups evaluated in DGM and in abnormal tissues like WMH and recent infarcts (i.e., patients who had hypertension vs. normotensive patients, patients who had lacunar vs. cortical strokes, patients with different PVS scores in the basal ganglia, and patients with different SVD scores). These differences were observed regardless of whether the parameters were obtained from images acquired before or 20 min after the contrast injection.

#### Hypertensive vs. Normotensive Patients

**Figure 5:**
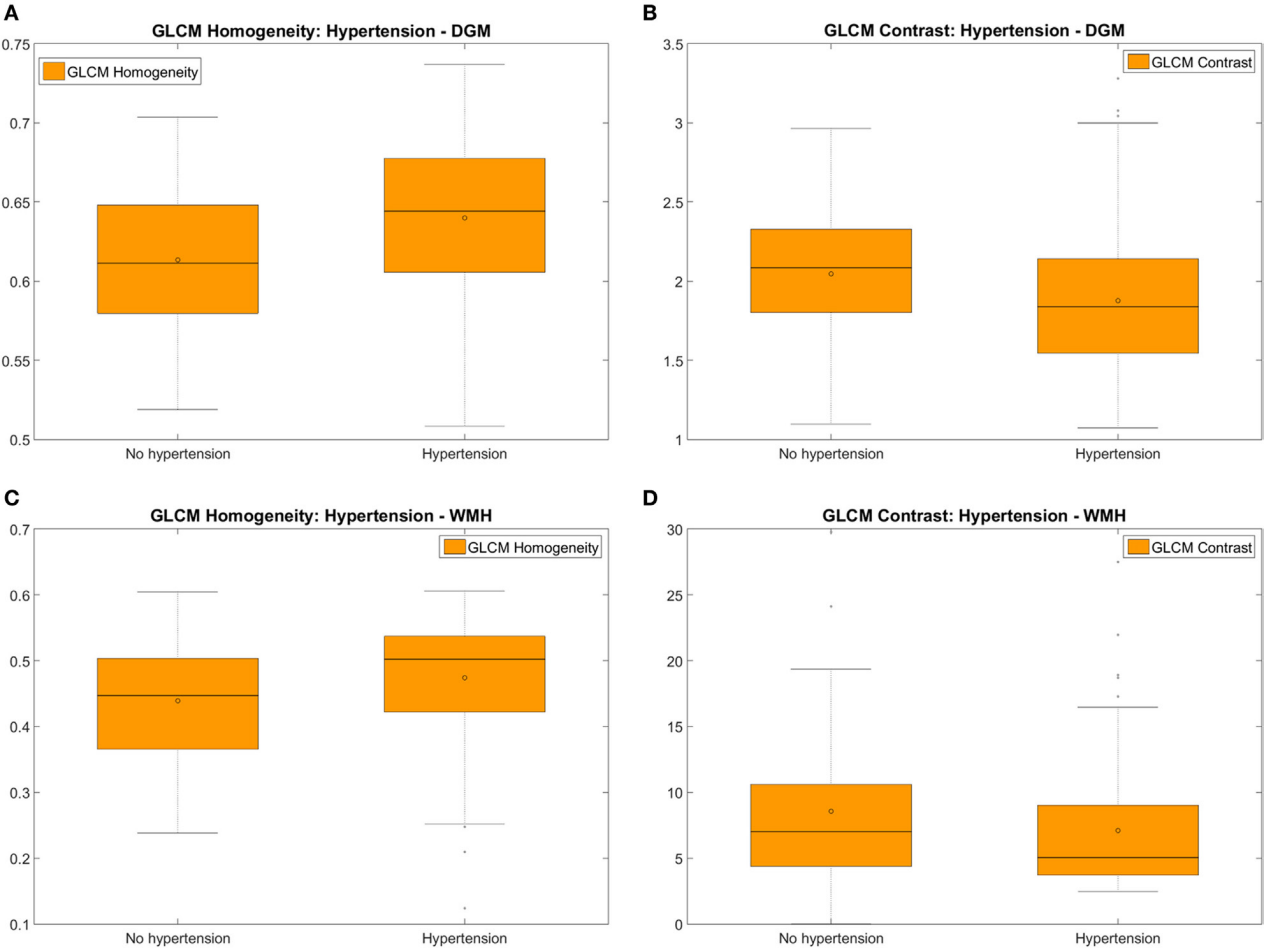
Measures of the pre-contrast FLAIR homogeneity **(A,C)** and variability (i.e. represented by the GLCM contrast) **(B,D)** of the textures corresponding to the deep gray matter (DGM) **(A,B)** and white matter hyperintensities (WMH) **(C,D)** of hypertensive vs. normotensive patients. Textures from post-contrast FLAIR images produced almost identical results.

With the exception of GLCM variance, all texture descriptors evaluated on DGM and WMH on pre- and post-contrast FLAIR images significantly differed between hypertensive and normotensive patients: they show more “homogeneity” in the tissues’ “texture” of FLAIR images from patients with hypertension than in those from normotensive patients. On NAWM, textural differences between these two groups of patients had the same pattern as on DGM and WMH, but were only significant on post-contrast images (*p* < 0.017). Table 4 and Figure 5 show the results for homogeneity and GLCM contrast. Values for the rest of the texture descriptors can be found in Data Sheet S2 in Supplementary Material (Table S2.2 and Figures S2.3 and S2.4 in Supplementary Material).

**Table 4:** Results of the significance (*p*-values) of the Kruskal–Wallis (K–W) and Mood’s Median tests for normotensive vs. hypertensive patients, patients grouped by scores of perivascular spaces in the basal ganglia and patients grouped by SVD scores 0–4.

			Cerebrospinal fluid		Normal-appearing white matter		Deep gray matter		Index stroke lesion		White matter hyperintensities	
					
			K–W	Median	K–W	Median	K–W	Median	K–W	Median	K–W	Median
Normotensive vs. hypertensive patients	Homogeneity	Pre-Gd	0.479	0.632	0.059	0.151	0.001**	0.002*	0.689	0.688	0.008*	0.007*
		Post-Gd	0.508	0.632	0.010*	0.038*	0.002*	0.017*	0.482	1.000	0.002*	0.007*
	Variability	Pre-Gd	0.807	0.873	0.087	0.264	0.005*	0.002*	0.415	0.422	0.033*	0.025*
		Post-Gd	0.662	0.632	0.013*	0.151	0.011*	0.017*	0.449	0.688	0.006*	0.011*

Patients grouped by BG-PVS scores	Homogeneity	Pre-Gd	0.014*	0.097	0.310	0.459	0.001*	0.014*	0.627	0.532	0.001**	0.001**
		Post-Gd	0.076	0.394	0.171	0.716	0.006*	0.072	0.680	0.615	0.001**	0.001**
	Variability	Pre-Gd	0.008*	0.013*	0.167	0.094	0.001**	0.002*	0.782	0.240	0.001**	0.001**
		Post-Gd	0.030*	0.026*	0.072	0.374	0.001**	0.010*	0.721	0.240	0.001**	0.001**

Patients grouped by SVD scores (0–4)	Homogeneity	Pre-Gd	0.002*	0.045*	0.149	0.167	0.001**	0.001*	0.291	0.362	0.001**	0.001**
		Post-Gd	0.001*	0.006*	0.122	0.073	0.004*	0.110	0.330	0.463	0.001**	0.001**
	Variability	Pre-Gd	0.001*	0.003*	0.018*	0.007*	0.001**	0.001**	0.192	0.021*	0.001**	0.001**
		Post-Gd	0.001**	0.001**	0.010*	0.013*	0.001**	0.001*	0.164	0.120	0.001**	0.001**

*p < 0.05.

**p < 0.001.

#### Lacunar vs. Cortical Ischemic Stroke Patients

On textural variability distribution (i.e., Kruskal–Wallis test), there were differences in the texture of the DGM between lacunar and cortical stroke patients, but only with borderline significance (*GLCM variance p* < 0.048 pre-contrast and *p* < 0.043 post-contrast): the texture in DGM was more “homogeneous” in patients with lacunar than cortical stroke, although the median values of the texture descriptors (i.e., from Mood’s Median test) did not differ significantly between these two patient groups. No significant differences between these two groups were observed in any of the texture descriptors measured in the NAWM or CSF.

In WMH, the medians of the *entropy* (pre- and post-contrast) (*p* < 0.014) were significantly higher in patients with lacunar than cortical ischemic stroke. The *entropy* distributions differed significantly between the groups (*p* < 0.023) on pre- and post-contrast images. However, not all texture descriptors that express variability in tissue behaved in the same way in the WMH regions: the median of the *GLCM variance* was higher in the lacunar group whereas the *GLCM contrast* was higher in the cortical group. A similar pattern was noted in the medians of the characteristics that express homogeneity: *GLCM correlation* and *homogeneity* were higher in the lacunar group whereas *energy* was higher in the cortical group (see Figure S2.6 in Supplementary Material). Nevertheless, these differences were not significant and disappeared on post-contrast images.

**Figure 6:**
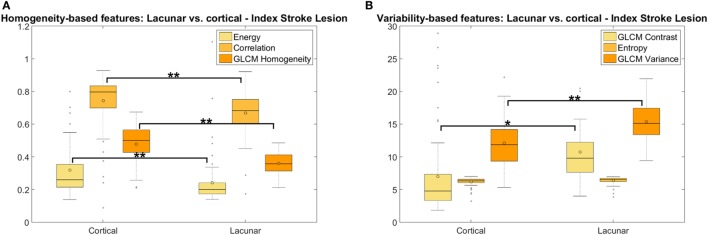
Measures of homogeneity **(A)** and variability **(B)** of the texture in the index stroke lesions between patients with lacunar and cortical ischemic stroke. The energy in panel **(A)** has been multiplied by 15 to give a similar range to the gray level co-occurrence matrix (GLCM) correlation and homogeneity, for visualization purposes (*p < 0.05; **p < 0.001). Median outputs from the six textural characteristics (i.e., three representing homogeneity and three representing variability in texture) are displayed grouped by cortical vs. lacunar, showing that texture in the index stroke lesion was more homogeneous in patients with cortical stroke rather than in those who had a lacunar stroke and that, consequently, heterogeneity was higher in lacunar than in cortical stroke lesions.

In index stroke lesions, the distributions and medians of all texture descriptors assessed pre- and post-contrast differed between the lacunar and cortical stroke patient groups (*energy, GLCM correlation, homogeneity, GLCM contrast*, and *GLCM variance*: *p* < 0.001, *entropy*: *p* < 0.006). As predicted, the medians of the texture descriptors that expressed “homogeneity” were significantly accentuated (i.e., had high values) if the stroke lesion was cortical and, in turn, more “heterogeneous” (textural variability indicators had higher values) if the stroke was of a lacunar type (Figure 6).

#### Patients with Different Basal Ganglia PVS Scores

**Figure 7:**
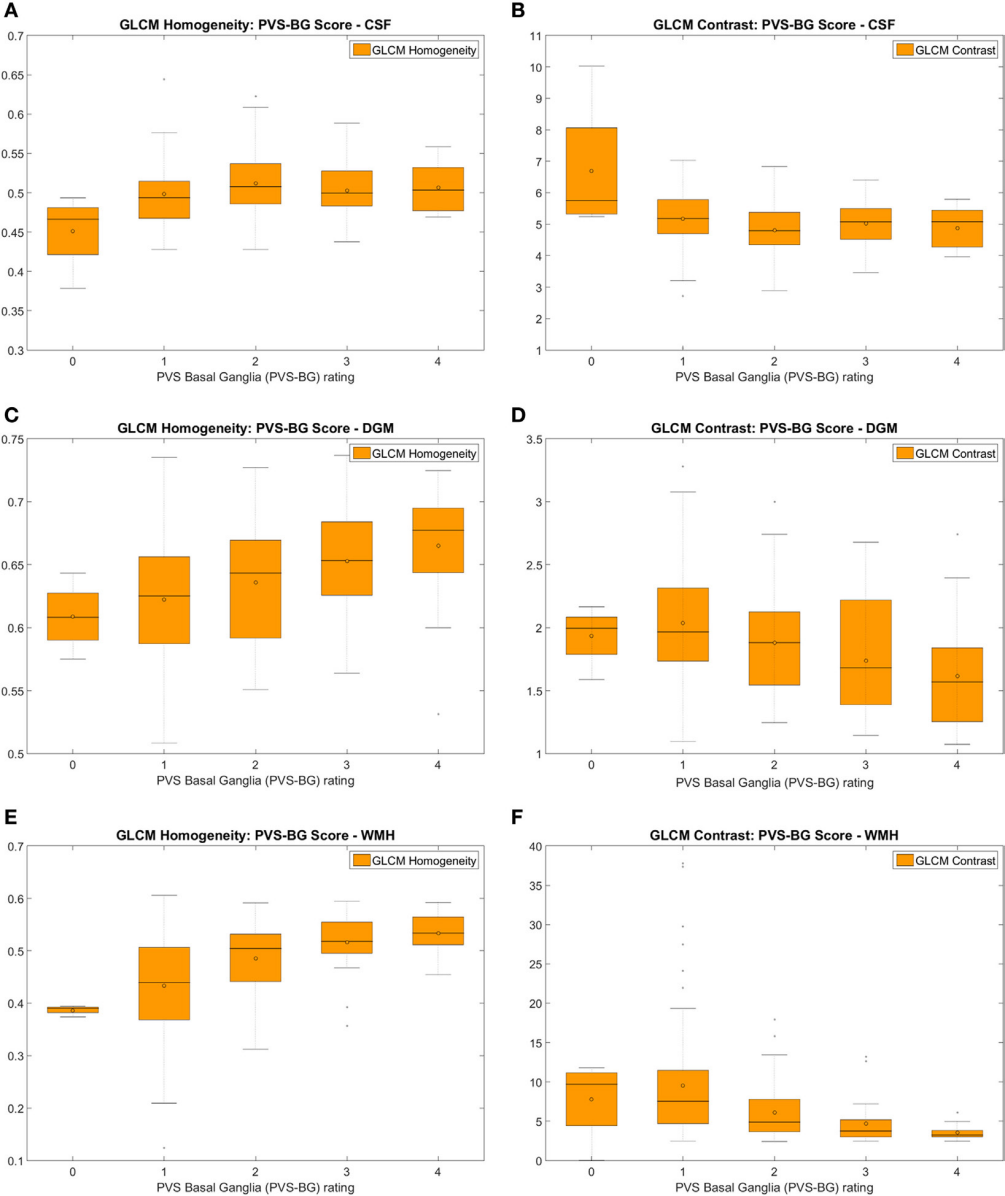
Measures of the homogeneity **(A,C,E)** and the variability (i.e. represented by the GLCM contrast) **(B,D,F)** of the textures corresponding to the cerebrospinal fluid (CSF) **(A,B)**, deep gray matter (DGM) **(C,D)**, and white matter hyperintensities (WMH) **(E,F)** of patients with different ratings of perivascular spaces (PVS) in the basal ganglia, obtained from pre-contrast FLAIR images. Similar results were obtained from post-contrast images.

Texture descriptors in CSF, DGM, and WMH differed significantly between patients grouped by PVS scores in the basal ganglia, showing increasing “homogeneity” with higher PVS scores. There were no statistically significant differences between these groups in the distributions or in the medians in the NAWM. In the index stroke lesions, *energy* (*p* < 0.046 pre-contrast and *p* < 0.015 post-contrast) and *entropy* (*p* < 0.019 post-contrast) significantly differed between the PVS groups. Table 4 shows the results from the Kruskal–Wallis and Mood’s Median tests for *GLCM homogeneity* and *GLCM contrast*. The ranges of the values of these two variables assessed in CSF, DGM, and WMH according to basal ganglia PVS scores ranging from 0 to 4 are illustrated in the boxplots of Figure 7.

#### Patients Grouped by SVD Scores

**Figure 8:**
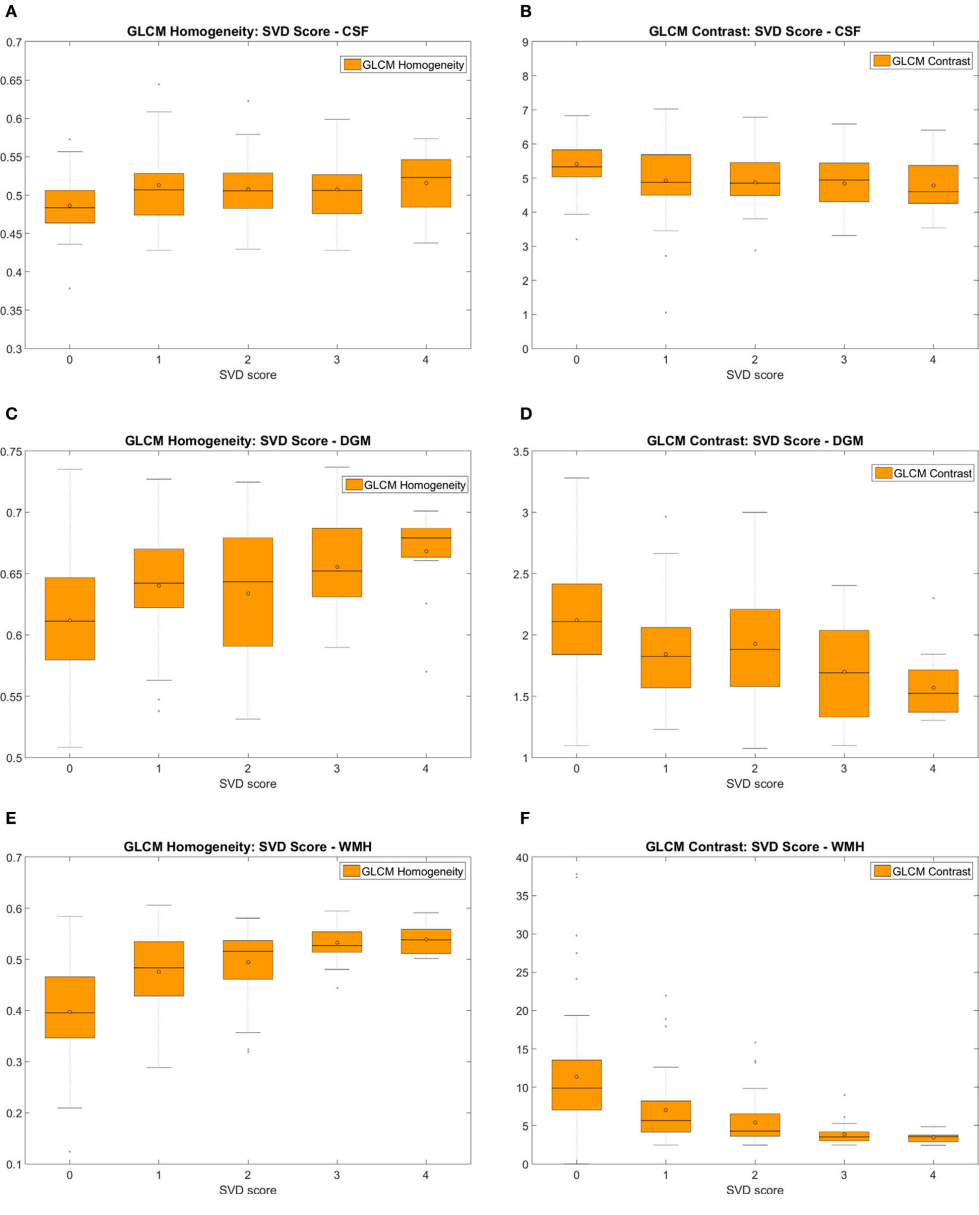

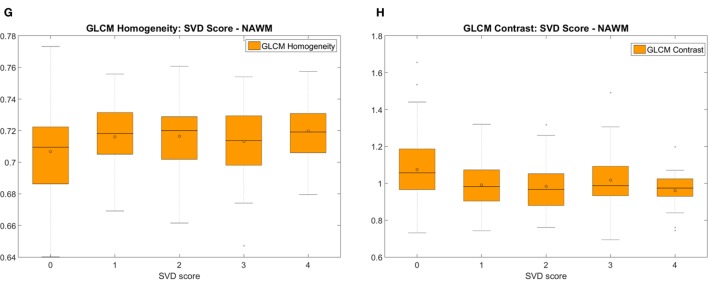
Measures of the pre-contrast FLAIR homogeneity **(A,C,E,G)** and the variability (i.e. represented by the GLCM contrast) **(B,D,F,H)** of the textures corresponding to the cerebrospinal fluid (CSF) **(A,B)**, deep gray matter (DGM) **(C,D)**, white matter hyperintensities (WMH) **(E,F)**, and normal-appearing white matter (NAWM) **(G,H)** of patients with different small vessel disease (SVD) scores. Similar patterns were obtained post-contrast.

Texture descriptors differed significantly in CSF, NAWM, DGM, and WMH between patients with low and high SVD scores. To illustrate these results, we selected *GLCM homogeneity* and *GLCM contrast* to represent, respectively, the “homogeneity” and “variability” of the texture in tissue (Table 4; Figure 8) (41). Boxplots (Figure 8) reveal that the texture was slightly more “homogeneous” as SVD score increased in all tissues in both pre- and post-contrast images. Also, there were marked pre-/post-contrast differences in the medians of the *energy* (*p* < 0.028) and the *GLCM variance* (*p* < 0.028) in the index stroke lesions, which are shown with the rest of the results, in the Data Sheet S2 in Supplementary Material.

### Validation of the Analysis

**Figure 9:**
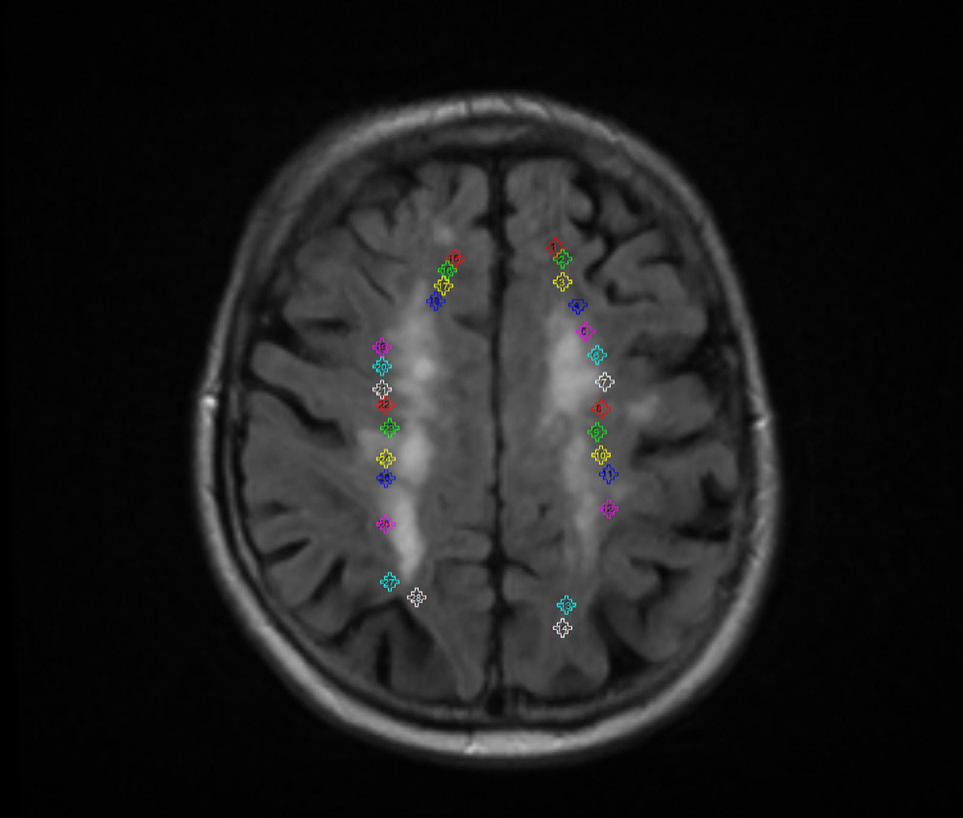
Example of the regions of interest manually placed to validate the analyses, evaluating the influence of the stroke lesion location/hemisphere in the outcome of the results.

In the ROIs manually placed as depicted in Figure 9, entropy values did not differ significantly between hemispheres (mean *Z* = −0.643, *p* = 0.52), and neither between ROIs (*n* = 39, χ^2^ = 2.16, *p* = 0.54) of the same patient.

## Discussion

In this paper, a meaningful subset of texture descriptors is used to shed light on the imaging characteristics of a neurological syndrome, in this case: SVD. To the best of our knowledge, this is the first time that texture analysis has been applied for this purpose on a relatively large sample (*n* = 204) with a wide range of SVD degree, as opposed to its well-known use in machine-learning classifiers in oncology (12, 23, 32). The results of the evaluation of the selected texture descriptors on tissues segmented on FLAIR images of mild stroke patients showed that the texture in normal tissues and WMH is more homogeneous in patients with increasing age, SVD features, and basal ganglia PVS scores, and in hypertension vs. normotension. This also confirms our hypothesis that with increasing age and SVD severity, already abnormal tissues become more uniformly abnormal (i.e., “homogenous”) while the differentiation between the “normal” and “abnormal” becomes less obvious.

As hypothesized, post-contrast texture change in CSF increased with respect to the pre-contrast texture with severity of white matter disease indicating more accumulation of gadolinium in CSF with increase of SVD burden. In general, the intravenous contrast seemed to affect more the texture in the tissues with more water content (i.e., more abnormal). From the clinical variables evaluated, Fazekas scores and age, known to be associated (51) and reported to be associated with increasing BBB permeability (6), predicted the post-contrast change in texture. These results indicate that, at macroscopic levels, the presence of the contrast agent in the central nervous system fluids as a consequence of an impaired BBB is more evident as the white matter burden is more abundant, thus confirming the usefulness of the texture analysis approach presented here in the study of the BBB.

It may seem a paradox that, with increasing age and presence of SVD indicators, the tissue texture becomes more “uniform” at the same time that the brain tissue becomes, visibly, more heterogeneous. In this approach, we are not extracting the texture descriptors from anatomically relevant regions (e.g., brain lobes, brain stem, and substantia nigra) or from the whole brain tissue. Instead, the texture descriptors are calculated in regions with similar pathophysiological characteristics (i.e., NAWM separately from WMH or recent stroke lesions, DGM, CSF), taking care that other imaging features that are evidence of disease (i.e., cavities, hemorrhages, etc.) were excluded from these tissue types. For example, regions visibly heterogeneous and difficult to characterize like old stroke lesions were separately masked out and excluded from the analysis. As previous study on white matter integrity in older brains suggests (37), in all tissues, the water mobility in the interstitial space increases with increasing pathological indicator scores (i.e., Fazekas, basal ganglia PVS, and SVD scores) perhaps in part because the interstitial space itself increases with tissue rarefaction (52).

All texture descriptors that express textural variability had higher median values in recent ischemic lesions if these were of lacunar type than cortical (before and after the intravenous contrast injection), possibly reflecting a degree of active microstructural flux strong enough to cause macrostructural changes perceivable even after considerably reducing the bit resolution of the MR image. Similarly, the median of the textural entropy in WMH, which expresses the randomness or disorder of the intensity levels in the region, was significantly higher in WMH of patients that had a stroke of lacunar type rather than in patients with cortical stroke, perhaps reflecting the higher SVD burden in lacunar stroke. WMH texture descriptors had similar tendencies to those measured in normal tissues when comparing groups of patients with different PVS and SVD scores, and patients with vs. without hypertension. However, this was not the case while comparing patients that had lacunar vs. cortical strokes: while WMH texture descriptors differed between lacunar and cortical groups, texture descriptors in normal tissues did not differ significantly between these groups of patients.

Tissue texture descriptors are not quantitative measurements of physiological BBB permeability indicators, but if sampled 20 min after intravenous contrast, the signal intensity changes in FLAIR MR images especially CSF are more likely to be indicative of a cumulative leakage in tissue (and CSF). The predictors of this textural change were SVD indicators previously related to subtle BBB dysfunction: age, hypertension and WMH burden represented by Fazekas scores (6, 33, 35, 53). Given the limitations of the existing DCE-MRI protocols for assessing BBB permeability without knowledge of vessel surface area (8, 9) alternative methods for assessing pre–post gadolinium differences as markers of leakage are worth pursuing. The fact that GLCM of *N* = 16, 32, and 64 yielded essentially the same results suggests that the method could be implemented much faster by quantizing the data with little change to the results. It might be that the key drivers of the textural features are large changes in signal intensity. So, when we take out the small changes (e.g., by quantization), we obtain similar results. Thus meaning that voxel size changes/differences would probably not influence the overall results. However, further tests on determining whether this type of analysis would be optimum for a certain “scale” are needed.

This study incorporates patients imaged from 1 to 3 months after presenting to clinic with stroke symptoms, where surely BBB breakdown and remodeling were underway. We evaluated whether our conclusions could have been affected by the global approach taken in the tissue analyses and evaluated interhemispheric textural differences in pairs of ROIs on similar anatomical regions, and differences in the textural variability between ROIs located in different arterial territories and distances with respect to the infarct. In agreement with previous studies (30, 54), the proximity/hemisphere of the infarcted region did not appear to have influenced our results.

This work has limitations. The results depend on the accuracy of previous tissue segmentation. We followed the image analysis protocol described in Ref. (43). Despite of its extensive validation and generalizability, it is recommended to always validate any segmentation protocol in the sample that the study uses. Furthermore, in this work only Haralick texture descriptors were analyzed. There are other texture descriptors that have proven to be more efficient and have better power in texture segmentation and classification schemes and may be useful for the discrimination of subtle tissue differences in SVD. For example, sub-band filtering in the Fourier domain with a second orientation pyramid and the use of local energy functions, granulometric size distributions and Gabor filters have all been used in medical imaging (55–57). However, their meaning is not intuitive, and as such, it is difficult to convey a clear message of the patterns of responses to the effects evaluated (i.e., burden of SVD, PVS, stroke subtype, and hypertension) on tissue images. The intuitiveness of the texture descriptors used here (i.e., not based on the results of the implementation of any dimensionality reduction method like principal component analysis, Fisher discriminant analysis, etc.) could be considered another limitation of this study. Future works should explore more texture descriptors followed by the application of a dimensionality reduction method.

## Ethics Statement

The study was approved by the Lothian Ethics of Medical Research Committee (REC 09/81101/54) and the NHS Lothian R+D Office (2009/W/NEU/14) and conducted according to the principles expressed in the Declaration of Helsinki.

## Author Contributions

MH: study conception and design, image processing, texture analysis, statistical analysis; wrote the manuscript. VG-C: texture analysis; participated in the study design; and wrote the manuscript. FC: statistical analysis; revised, edited, and approved the manuscript. ES: image processing; revised and approved the manuscript. SM: patient recruitment; clinical patient assessment; and revised, edited, and approved the manuscript. PA: image protocol design and image quality control and supervision; revised, edited, and approved the manuscript. WN: texture analysis; revised, edited, and approved the manuscript. JW: principal investigator; radiological assessment; participated in the study design; and revised, edited, and approved the manuscript.

## Conflict of Interest Statement

The authors declare that the research was conducted in the absence of any commercial or financial relationships that could be construed as a potential conflict of interest. The reviewer, ME, and handling editor declared their shared affiliation, and the handling editor states that the process nevertheless met the standards of a fair and objective review.

## Footnotes

^1^http://hdl.handle.net/10283/2718.

^2^www.sourceforge.com/projects/bric1936.

^3^http://fsl.fmrib.ox.ac.uk/fsl/fslwiki/.

^4^www.sas.com.

## Abbreviations

GLCM, gray level co-occurrence matrix; BBB, blood–brain barrier; BG-PVS, score of perivascular spaces in the basal ganglia; SVD, small vessel disease; WMH, white matter hyperintensities; NAWM, normal-appearing white matter; CSF, cerebrospinal fluid; DGM, deep gray matter; FLAIR, fluid-attenuation inversion recovery; DCE-MRI, dynamic contrast-enhanced magnetic resonance imaging; IQR, interquartile range; CI, confidence interval.

## References

[1] Wardlaw JM Smith C Dichgans M. Mechanisms of sporadic cerebral small vessel disease: insights from neuroimaging. Lancet Neurol (2013) 12:483–97. 10.1016/S1474-4422(13)70060-7 23602162 PMC3836247

[2] Topakian R Barrick TR Howe FA Markus HS. Blood-brain barrier permeability is increased in normal-appearing white matter in patients with lacunar stroke and leucoaraiosis. J Neurol Neurosurg Psychiatry (2010) 81:192–7. 10.1136/jnnp.2009.172072 19710048

[3] Farrall AJ Wardlaw JM Blood brain barrier: ageing and microvascular disease – systemic review and meta-analysis. Neurobiol Aging (2009) 30:337–52. 10.1016/j.neurobiolaging.2007.07.015 17869382

[4] Uh J Yezhuvath U Cheng Y Lu H. In vivo vascular hallmarks of diffuse leukoaraiosis. J Magn Reson Imaging (2010) 32:184–90. 10.1002/jmri.22209 20578025 PMC3236451

[5] Hainsworth AH Markus HS. Do in vivo experimental models reflect human cerebral small vessel disease? A systematic review. J Cereb Blood Flow Metab (2008) 28:1877–91. 10.1038/jcbfm.2008.91 18698331

[6] Wardlaw JM Makin S Valdes-Hernandez MC Armitage PA Heye AK Chappell FM Blood-brain barrier failure as a core mechanism in cerebral small vessel disease and dementia: evidence from a cohort study. Alzheimers Dement (2017) 13(6):634–43. 10.1016/j.jalz.2016.09.006

[7] Heye AK Culling RD Valdes Hernandez MC Thrippleton MJ Wardlaw JM Assessment of blood-brain barrier disruption using dynamic contrast-enhanced MRI. A systematic review. Neuroimage Clin (2014) 6:262–74. 10.1016/j.nicl.2014.09.002 25379439 PMC4215461

[8] Armitage PA Farrall AJ Carpenter TK Doubal FN Wardlaw JM. Use of dynamic contrast-enhanced MRI to measure subtle blood-brain barrier abnormalities. Magn Reson Imaging (2011) 29:305–14. 10.1016/j.mri.2010.09.002 21030178 PMC4025605

[9] Heye AK Thrippleton MJ Armitage PA Valdes Hernandez MC Makin SD Glatz A Tracer kinetic modelling for DCE-MRI quantification of subtle blood-brain barrier permeability. Neuroimage (2015) 125:446–55. 10.1016/j.neuroimage.2015.10.018 26477653 PMC4692516

[10] Wardlaw JM Doubal F Armitage P Chappell F Carpenter T Maniega SM Lacunar stroke is associated with diffuse blood-brain barrier dysfunction. Ann Neurol (2009) 65:194–202. 10.1002/ana.21549 19260033

[11] Kidwell CS Burgess R Menon R Warach S Latour LL. Hyperacute injury marker (HARM) in primary hemorrhage: a distinct form of CNS barrier disruption. Neurology (2011) 77:1725–8. 10.1212/WNL.0b013e318236ef46 22031531 PMC3208951

[12] Agner SC Soman S Libfield E McDonald M Thomas K Englander S Textural kinetics: a novel dynamic contrast-enhanced (DCE)-MRI feature for breast lesion classification. J Digit Imaging (2011) 24:446–63. 10.1007/s10278-010-9298-1 20508965 PMC3092055

[13] Alic L van Vliet M van Dijke CF Eggermont AM Veenland JF Niessen WJ. Heterogeneity in DCE-MRI parametric maps: a biomarker for treatment response? Phys Med Biol (2011) 56:1601–16. 10.1088/0031-9155/56/6/006 21335648

[14] Chaudhury B Zhou M Goldgof DB Hall LO Gatenby RA Gillies RJ Heterogeneity in intratumoral regions with rapid gadolinium washout correlates with estrogen receptor status and nodal metastasis. J Magn Reson Imaging (2015) 42(5):1421–30. 10.1002/jmri.24921 25884277 PMC5017794

[15] Eliat P-A Damien O Saikali S Carsin B Saint-Jalmes H De Certaines J Can dynamic contrast-enhanced magnetic resonance imaging combined with texture analysis differentiate malignant glioneuronal tumors from other glioblastoma? Neurol Res Int (2012) 2012:195176 10.1155/2012/195176 22203901 PMC3238409

[16] Kale MC Fleig JD Imal N Assessment of feasibility to use computer aided texture analysis based tool for parametric images of suspicious lesions in DCE-MR mammography. Comput Math Methods Med (2013) 2013:872676 10.1155/2013/872676 23653668 PMC3638704

[17] Karahaliou A Vassiou K Arikidis NS Skiadopoulos S Kanavou T Costaridou L. Assessing heterogeneity of lesion enhancement kinetics in dynamic contrast-enhanced MRI for breast cancer diagnosis. Br J Radiol (2010) 83:296–306. 10.1259/bjr/50743919 20335440 PMC3473457

[18] Nie K Chen JH Yu HJ Chu Y Nalcioglu O Su MY. Quantitative analysis of lesion morphology and texture features for diagnostic prediction in breast MRI. Acad Radiol (2008) 15:1513–25. 10.1016/j.acra.2008.06.005 19000868 PMC2791407

[19] Rose CJ Mills SJ O’Connor JP Buonaccorsi GA Roberts C Watson Y Quantifying spatial heterogeneity in dynamic contrast-enhanced MRI parameter maps. Magn Reson Med (2009) 62:488–99. 10.1002/mrm.22003 19466747

[20] Soares F Janela F Pereira M Seabra J Freire MM Classification of breast masses on contrast-enhanced magnetic resonance images through log detrended fluctuation cumulant-based multifractal analysis. IEEE Syst J (2014) 8:929–38. 10.1109/JSYST.2013.2284101

[21] Wang TC Huang YH Huang CS Chen JH Huang GY Chang YC Computer-aided diagnosis of breast DCE-MRI using pharmacokinetic model and 3-D morphology analysis. Magn Reson Imaging (2014) 32:197–205. 10.1016/j.mri.2013.12.002 24439361

[22] Woods BJ Clymer BD Kurc T Heverhagen JT Stevens R Orsdemir A Malignant-lesion segmentation using 4D co-occurrence texture analysis applied to dynamic contrast-enhanced magnetic resonance breast image data. J Magn Reson Imaging (2007) 25:495–501. 10.1002/jmri.20837 17279534

[23] Jianhua Y Chen J Chow C Breast tumor analysis in dynamic contrast enhanced MRI using texture features and wavelet transform. IEEE J Sel Top Signal Process (2009) 3:94–100. 10.1109/JSTSP.2008.2011110

[24] Ahmed A Gibbs P Pickles M Turnbull L. Texture analysis in assessment and prediction of chemotherapy response in breast cancer. J Magn Reson Imaging (2013) 38:89–101. 10.1002/jmri.23971 23238914

[25] Golden DI Lipson JA Telli ML Ford JM Rubin DL. Dynamic contrast-enhanced MRI-based biomarkers of therapeutic response in triple-negative breast cancer. J Am Med Inform Assoc (2013) 20:1059–66. 10.1136/amiajnl-2012-001460 23785100 PMC3822111

[26] Teruel JR Heldahl MG Goa PE Pickles M Lundgren S Bathen TF Dynamic contrast-enhanced MRI texture analysis for pretreatment prediction of clinical and pathological response to neoadjuvant chemotherapy in patients with locally advanced breast cancer. NMR Biomed (2014) 27:887–96. 10.1002/nbm.3132 24840393

[27] Torheim T Malinen E Kvaal K Lyng H Indahl UG Andersen EK Classification of dynamic contrast enhanced MR images of cervical cancers using texture analysis and support vector machines. IEEE Trans Med Imaging (2014) 33:1648–56. 10.1109/TMI.2014.2321024 24802069

[28] Chen W Giger ML Li H Bick U Newstead GM. Volumetric texture analysis of breast lesions on contrast-enhanced magnetic resonance images. Magn Reson Med (2007) 58:562–71. 10.1002/mrm.21347 17763361

[29] Viswanath S Bloch N Rofsky N Lenkinski R Genega E Chappelow J Registration, and cancer detection scheme on 3 tesla in vivo prostate DCE MRI. Med Image Comput Comput Assist Interv (2008) 11:662–9. 18979803 10.1007/978-3-540-85988-8_79 PMC2810962

[30] Viksne L Valdés Hernández MC Hoban K Heye AK Gonzalez-Castro V Wardlaw JM In: Tryphon L Xujiong Y, editors. Textural Characterisation on Regions of Interest: A Useful Tool for the Study of Small Vessel Disease. UK: University of Lincoln (2015). p. 66–71. Available from: www.miua.org.uk

[31] Haralick RM Shanmugam K Dinstein I Textural features for image classification. IEEE Trans Syst Man Cybern (1973) SMC-3:610–21. 10.1109/TSMC.1973.4309314

[32] Cai H Liu L Peng Y Wu Y Li L. Diagnostic assessment by dynamic contrast-enhanced and diffusion-weighted magnetic resonance in differentiation of breast lesions under different imaging protocols. BMC Cancer (2014) 14:366. 10.1186/1471-2407-14-366 24885156 PMC4036635

[33] Wardlaw JM Doubal FN Valdes-Hernandez MC Wang X Chappell FM Shuler K Blood-brain barrier permeability and long term clinical and imaging outcomes in cerebral small vessel disease. Stroke (2013) 44:525–7. 10.1161/STROKEAHA.112.669994 23233386 PMC3843346

[34] Wardlaw JM Differing risk factors and outcomes in ischemic stroke subtypes: focus on lacunar stroke. Future Neurol (2011) 6:201–21. 10.2217/fnl.11.1

[35] Heye AK Thrippleton MJ Chappell FM Valdes Hernandez MC Armitage PA Makin SD Blood pressure and sodium: association with MRI markers in cerebral small vessel disease. J Cereb Blood Flow Metab (2016) 36(1):264–74. 10.1038/jcbfm.2015.64 25899292 PMC4758556

[36] Doubal FN MacLullich AM Ferguson KJ Dennis MS Wardlaw JM. Enlarged perivascular spaces on MRI are a feature of cerebral small vessel disease. Stroke (2010) 41:450–4. 10.1161/STROKEAHA.109.564914 20056930

[37] Munoz Maniega S Valdes Hernandez M Clayden JD Royle NA Murray C Morris Z White matter hyperintensities and normal-appearing white matter integrity in the aging brain. Neurobiol Aging (2015) 36(2):909–18. 10.1016/j.neurobiolaging.2014.07.048 25457555 PMC4321830

[38] de Groot M Cremers LGM Ikram MA Hofman A Krestin GP van der Lugt A White matter degeneration with aging: longitudinal diffusion MR imaging analysis. Radiology (2015) 279(2):532–41. 10.1148/radiol.2015150103 26536311

[39] Simpson JE Wharton SB Cooper J Gelsthorpe C Baxter L Forster G Alterations of the blood-brain barrier in cerebral white matter lesions in the ageing brain. Neurosci Lett (2010) 486:246–51. 10.1016/j.neulet.2010.09.063 20887772

[40] Fazekas F Barkhof F Wahlund LO Pantoni L Erkinjuntti T Scheltens P CT and MRI rating of white matter lesions. Cerebrovasc Dis (2003) 13:31–6. 10.1159/000049147 11901240

[41] Huijts M Duits A van Oostenbrugge RJ Kroon AA de Leeuw PW Staals J. Accumulation of MRI markers of cerebral small vessel disease is associated with decreased cognitive function. A study in first-ever lacunar stroke and hypertensive patients. Front Aging Neurosci (2013) 5:72. 10.3389/fnagi.2013.00072 24223555 PMC3818574

[42] Staals J Makin SD Doubal F Dennis M Wardlaw JM Stroke subtype, vascular risk factors and total MRI brain small vessel disease burden. Neurology (2014) 83:1228–34. 10.1212/WNL.0000000000000837 25165388 PMC4180484

[43] Valdes Hernandez MC Armitage PA Thrippleton MJ Chappell F Sandeman E Munoz Maniega S Rationale, design and methodology of the image analysis protocol for studies of patients with cerebral small vessel disease and mild stroke. Brain Behav (2015) 5(12):e00415. 10.1002/brb3.415 26807340 PMC4714639

[44] Wardlaw JM Chappell FM Valdés Hernández MC Makin SD Staals J Shuler K White matter hyperintensity reduction and outcomes after minor stroke. Neurology (2017) (in press). 10.1212/WNL.0000000000004328 PMC5589793 28794252

[45] Hernandez MC Ferguson KJ Chappell FM Wardlaw JM. New multispectral MRI data fusion technique for white matter lesion segmentation: method and comparison with thresholding in FLAIR images. Eur Radiol (2010) 20:1684–91. 10.1007/s00330-010-1718-6 20157814 PMC2882045

[46] Smith SM Brady JM SUSAN – a new approach to low level image processing. Int J Comp Vis (1997) 23:45–78. 10.1023/A:1007963824710

[47] Patenaude B Smith SM Kennedy DN Jenkinson M. A Bayesian model of shape and appearance for subcortical brain segmentation. Neuroimage (2011) 56:907–22. 10.1016/j.neuroimage.2011.02.046 21352927 PMC3417233

[48] Jenkinson M Bannister P Brady M Smith S. Improved optimization for the robust and accurate linear registration and motion correction of brain images. Neuroimage (2002) 17:825–41. 10.1006/nimg.2002.1132 12377157

[49] Farrell C Chappell F Armitage PA Keston P MacLullich A Shenkin S Development and initial testing of normal reference MR images for the brain at ages 65–70 and 75–80 years. Eur Radiol (2009) 19:177–83. 10.1007/s00330-008-1119-2 18690455

[50] Vickers AJ The use of percentage change from baseline as an outcome in a controlled trial is statistically inefficient: a simulation study. BMC Med Res Methodol (2001) 1:6 10.1186/1471-2288-1-6 11459516 PMC34605

[51] Munoz Maniega S Chappell FM Valdes Hernandez MC Armitage PA Makin SD Haye AK Integrity of normal-appearing white matter: influence of age, visible lesion burden and hypertension in patients with small-vessel disease. J Cereb Blood Flow Metab (2017) 37(2):644–56. 10.1177/0271678X16635657 26933133 PMC5381455

[52] van Walderveen MA Kamphorst W Scheltens P van Waesberghe JH Ravid R Valk J Histopathologic correlate of hypointense lesions on T1-weighted spin-echo MRI in multiple sclerosis. Neurology (1998) 50:1282–8. 10.1212/WNL.50.5.1282 9595975

[53] Doubal FN Dennis MS Wardlaw JM Lacunar stroke is associated with increased blood brain barrier permeability. Cerebrovasc Dis (2008) 25(Suppl 2):125 10.1159/000132090

[54] Valdes Hernandez MC Qiu X Wang X Wiseman S Sakka E Maconick LC Interhemispheric characterization of small vessel disease imaging markers after subcortical infarct. Brain Behav (2016) 7:e00595. 10.1002/brb3.595 28127514 PMC5256179

[55] Reyes-Aldasoro CC Multiresolution Volumetric Texture Segmentation [Dissertation]. University of Warwick (2004) 1–148.

[56] Chen Y Gray-scale morphological granulometric texture classification. Opt Eng (1994) 33:2713–22. 10.1117/12.173552

[57] Materka A Strzelecki M Texture Analysis Methods – A Review. Technical University of Lodz, Institute of Electronics, COSTB11 (1998). p. 1–33.

