# Supplementary material for: Application of Texture Analysis to Study Small Vessel Disease and Blood–Brain Barrier Integrity
Source: Front Neurol. 2017 Jul 19;8:327. doi: 10.3389/fneur.2017.00327 (PMC5515862; doi:10.3389/fneur.2017.00327)
Supplement: Supplementary file 1 [file Data_Sheet_1.DOCX]

**Application of texture analysis to study small vessel disease and blood brain barrier integrity**

**Supplementary Material 1 – Textural descriptors and variance-to-mean intensity ratios**

The co-occurrence matrix

We use second order statistics computed from a grey-level co-occurrence matrix (GLCM) [1], extracted as per Figure S1.1.


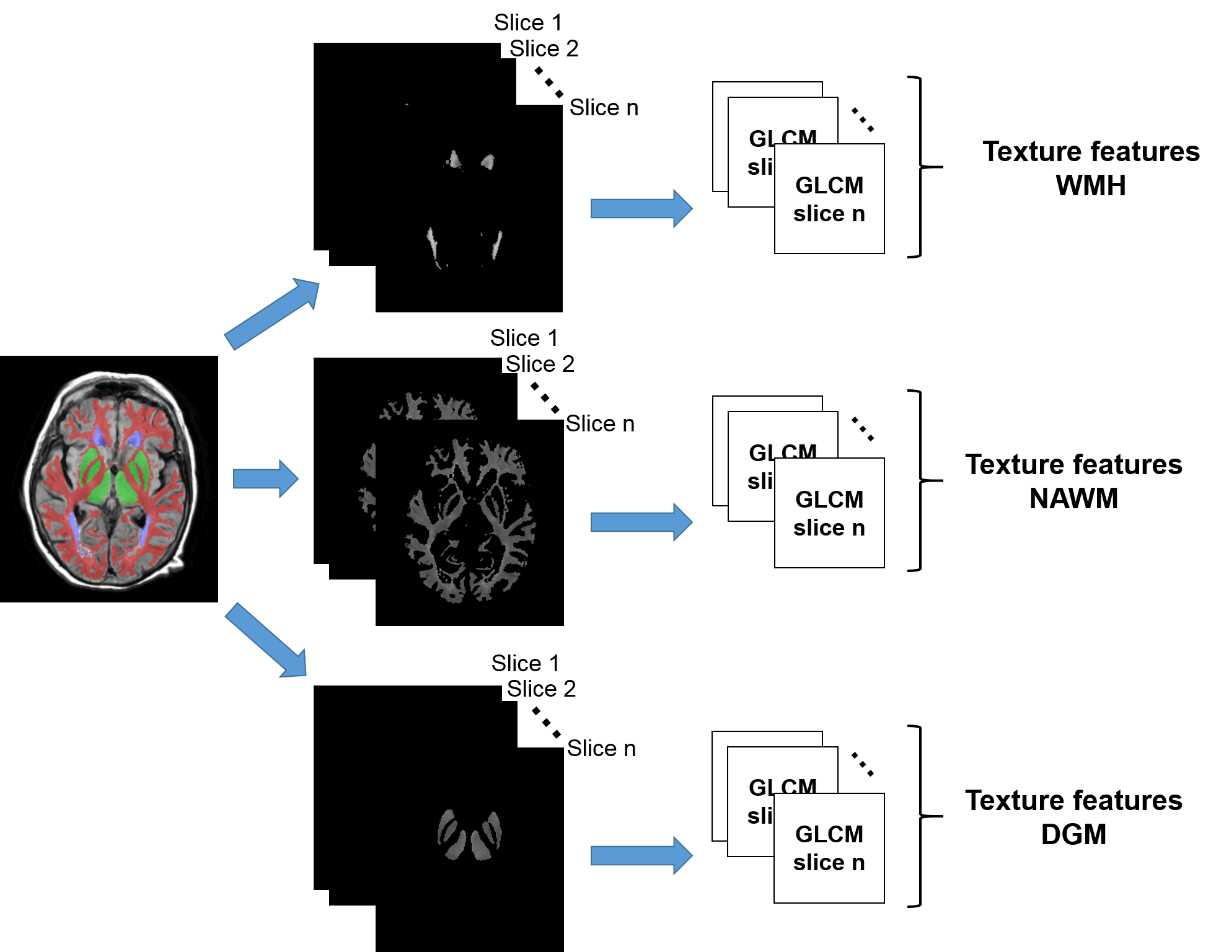


**Figure S1.1.** Process followed to obtain the texture features (i.e. texture descriptors) of the white matter hyperintensities (WMH), normal white matter (NAWM) and deep grey matter (DGM) (from top to bottom) in the FLAIR images. The process for the cerebrospinal fluid (CSF) and Index Stroke Lesions is the same.

The GLCM is a matrix defined to express the spatial relationship between pairs of pixels in an image, ultimately reflecting the distribution of the co-occurrence of pairs of pixels, where one is called reference and the other is called neighbour, at a given offset (i.e., a distance $d$ and an orientation $\theta$).

Let *P* be a matrix with size *N*x*N*, whose elements $P(i,j)$ are the number of pairs of pixels in the image with grey levels $i$ and $j$ that are separated by a line of length $d$ and angle $\theta$ with respect to the reference direction, which is usually the horizontal. Petrou and Garcia-Sevilla [2] defined the GLCM as:

| ${P(i,j)}_{d,\theta}=\sum_{k=1}^{n} \sum_{l=1}^{m} \delta\left[ i-f(k,l) \right]\delta\left[ j-f(k+d\cos\theta,l+d\sin\theta) \right]$, | (1) |
| --- | --- |

where $n$ and $m$ are the number of rows and columns of the image, respectively; $f(k,l)$ is the intensity of the pixel in the position $\left( k,l \right)$ of the image and $\delta(a-b)$ is a function which yields a value of 1 if $a=b$ or 0 otherwise. Figure S1.1 shows an example of the computation of two GLCMs on an image after the region of interest is extracted. Notice that the image space not occupied by the region of interest (i.e. tissue of certain type) is transformed into NaN (i.e. **N**ot **a** **N**umber). This is to avoid distortion in the calculations caused by the image background.


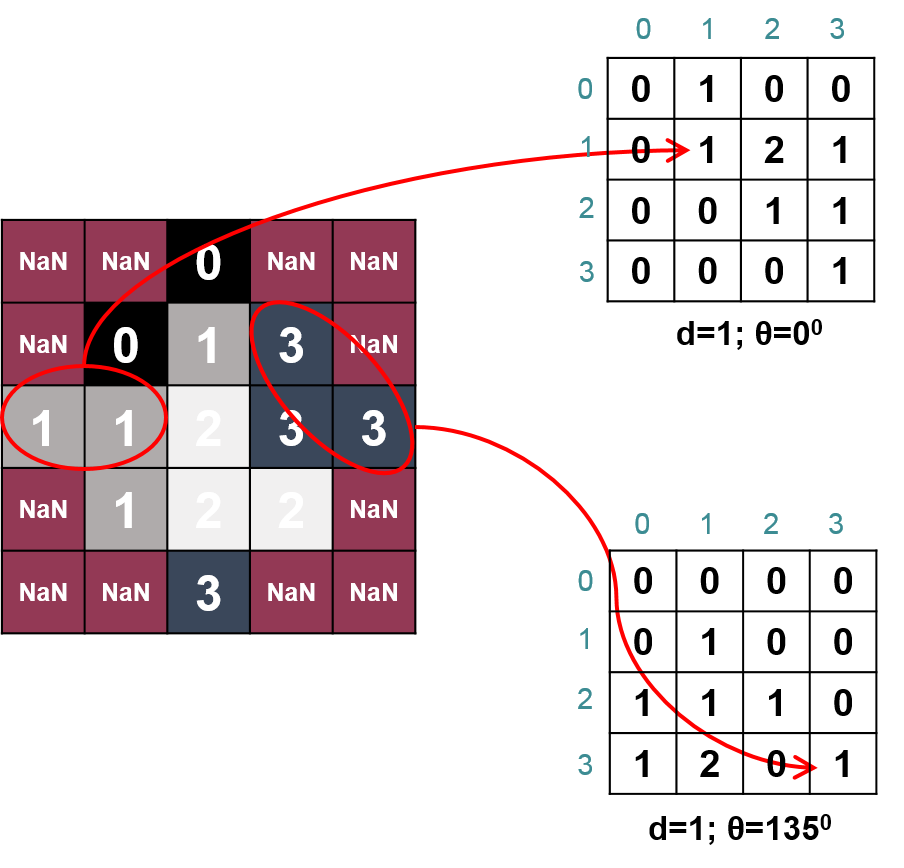


**Figure S1.2.** Example of the calculation of two co-occurrence matrices on an image

Second order statistics extracted from the co-occurrence matrix

Let $N$ be the number of different grey levels into which the texture intensities are quantised; $p(i,j)$ be the $(i,j)$-th entry in the normalised GLCM; and $p_{x}(i)$ (respectively $p_{y}(i)$) be the i-th entry in the marginal probability matrix obtained by summing the rows (and columns) of $p(i,j)$.

1. Contrast (referred to as “GLCM contrast” to differentiate it from the intravenous contrast) is a measure of intensity variations between the reference pixel and its neighbour. The higher this value, the higher the variation of the intensities within the texture. Defined as:

| $GLCM contrast=\sum_{n=0}^{N} n^{2}p_{x-y}(n)$ | (2) |
| --- | --- |

where $p_{x-y}=\sum_{i} \sum_{j} p(i,j)$, with $\left| i-j \right|=n$ where $n=0,1,\ldots,N-1$.

1. Correlation (referred to as “GLCM correlation”) measures the linear dependency of intensities in the GLCM. It presents how a reference pixel is related to its neighbour: 0 means uncorrelated and 1 means perfectly correlated. It is defined as:

| $GLCM correlation=\frac{\sum_{i} \sum_{j} ijp\left( i,j \right)-\mu_{x}\mu_{y}}{\sigma_{x}\sigma_{y}}$ | (3) |
| --- | --- |

where µ_x_ (resp. µ_y_) and σ_x_ (resp. σ_y_) are the mean and standard deviation of p_x_ (resp. p_y_), respectively.

1. Inverse difference moment (Homogeneity) measures the local homogeneity of a texture. It is higher when the distribution of the GLCM elements is close to the GLCM diagonal, i.e., when the intensities of the texture are similar (i.e. the tissue/ROI is homogeneous).

| $Homogeneity=\sum_{i} \sum_{j} \frac{1}{1+\left( i-j \right)^{2}}p(i,j)$ | (4) |
| --- | --- |

1. Angular second moment (Energy) measures the uniformity of a texture. When intensities within an ROI are very similar, this value will be large.

| $Energy=\sum_{i} \sum_{j} {p(i,j)}^{2}$ | (5) |
| --- | --- |

1. Sum of squares: Variance (referred to as “GLCM variance”) is a measure of the dispersion of the values around the mean of combinations of reference and neighbourhood pixels. The higher this value, the less homogeneous the texture of the tissue/ROI will be.

| $GLCM variance=\sum_{i} \left( i-\mu_{x} \right)^{2}p_{x}(i)$ | (6) |
| --- | --- |

1. Entropy is an expression of the chaos or disorder (i.e. randomness) present in that texture. Its meaning is similar to that of the GLCM variance, but the equation used to calculate it differs:

| $Entropy=\sum_{i} \sum_{j} p\left( i,j \right)\log(p(i,j))$ | (7) |
| --- | --- |

First vs. second order statistical descriptors. Entropy analysis on synthetic images

First order statistical (i.e. histogram-based) textural descriptors only use information about the distribution of grey level intensities, which is given by the image histogram. The information about the relative position of the “dark” and “bright” pixels is a valuable information that is lost when descriptors are computed upon the histogram. As an example, we analysed the results from calculating the entropy as a first and a second order statistic on two synthetic images. The images from Figure S1.2 have been generated using Matlab R2014a to have exactly the same number of pixels on four different grey levels as their histograms show. The only difference between both images is the spatial distribution of the pixels, resulting in texture A and B as shown in the upper row of Figure S1.3.


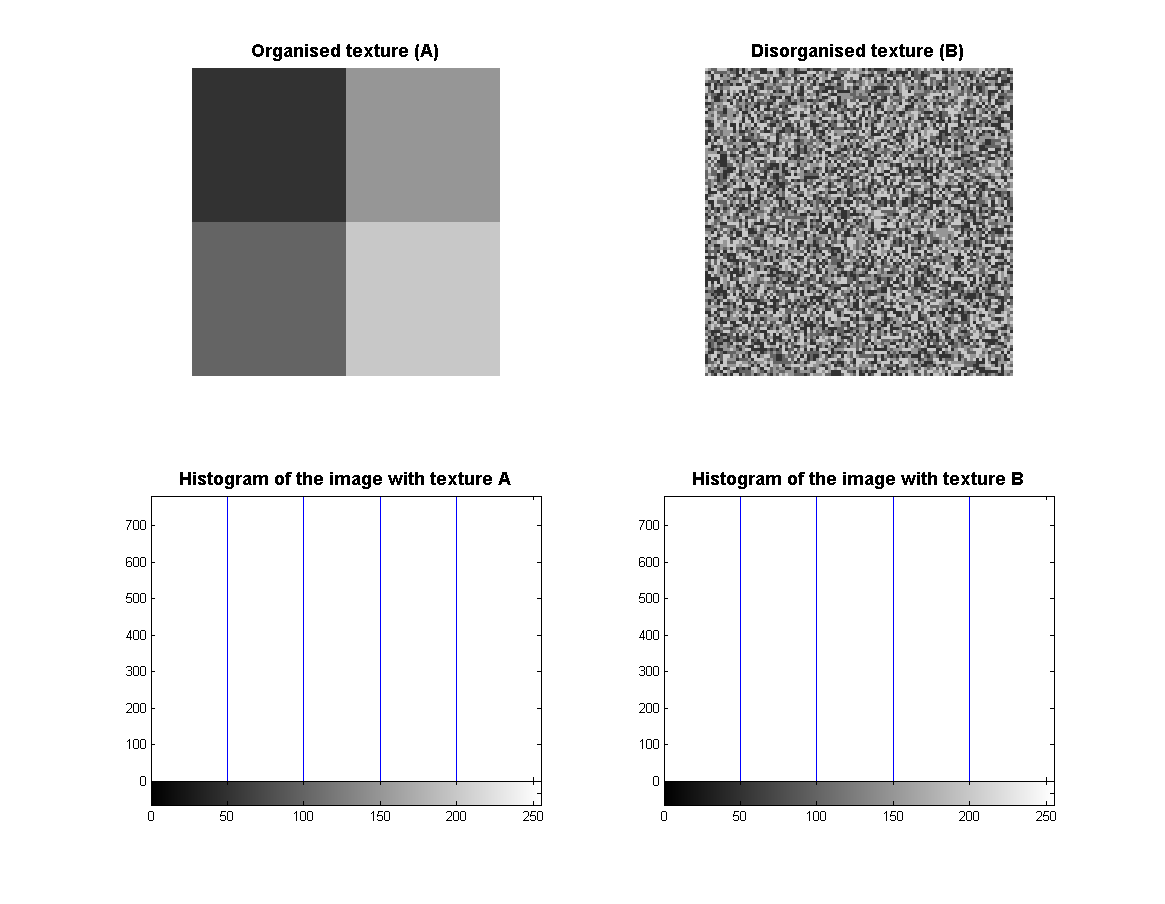


**Figure S1.3.** Synthetic images exhibiting the same grey levels and different textures (above) and their histograms (below)

When we calculated the entropy as:

| $Entropy=\sum p\left( \log p \right)^{2}$ | (8) |
| --- | --- |

considering *p* as the histogram counts (i.e. Matlab function “entropy”), both textures had the same entropy, equal to 2.

We also calculated the entropy using GLCMs for distances d=1, 2 and 3. For each distance, we computed four GLCMs: for four different orientations: θ=0, 45, 90 and 135 degrees. The final entropy for each distance was obtained averaging the entropies calculated from the four different orientations. The results are shown in Table 1.

Table 1. Entropy of the GLCMs of the synthetic images

|  | θ | **Entropy in Texture A GLCM_d,θ_** | **Entropy in Texture B GLCM_d,θ_** |
| --- | --- | --- | --- |
| d=1 | 0 | 2.0714 | 3.9993 |
|  | 45 | 2.1427 | 3.9994 |
|  | 90 | 2.0714 | 3.9991 |
|  | 135 | 2.1427 | 3.9993 |
|  | Mean θ | 2.1070 | 3.9993 |
| d=2 | 0 | 2.1233 | 3.9993 |
|  | 45 | 2.2466 | 3.9996 |
|  | 90 | 2.1233 | 3.9991 |
|  | 135 | 2.2466 | 3.9993 |
|  | Mean θ | 2.1850 | 3.9993 |
| d=3 | 0 | 2.1681 | 3.9998 |
|  | 45 | 2.3362 | 3.9995 |
|  | 90 | 2.1681 | 3.9994 |
|  | 135 | 2.3362 | 3.9990 |
|  | Mean θ | 2.2521 | 3.9994 |

Entropies calculated from the GLCMs (i.e. second order statistical descriptors) capture the textural difference between the two images: Texture B is much more “disordered” than Texture A but this disorder is invariant to the relative distance between pixel pairs and their relative orientation, as perceived from Figure S1.3.

Variance-to-mean intensity ratios as surrogate measures to evaluate pre-/post-contrast change

Differences between the variance-to-mean intensity ratios (VMR) measured in a region of interest (ROI) on the post-contrast FLAIR images and the VMR in the same regions on the pre-contrast FLAIR images express how much the proportion of the dispersion of the intensities of the ROI with respect to their mean value change after the injection of the intravenous agent, giving an idea of the effect of the contrast agent in the intensities of the ROI. They are defined as:

| $\Delta VMR=\frac{\sigma_{post-contrast}^{2}}{\mu_{post-contrast}}-\frac{\sigma_{pre-contrast}^{2}}{\mu_{post-contrast}}$ | (9) |
| --- | --- |

where σ is the standard deviation and µ is the mean of the intensity levels in the ROI.

**References**

[1] Haralick RM, Shanmugam K, Dinstein I. Textural Features for Image Classification. IEEE Trans Sys Man Cyber 1973, 3(6):610-621.

[2] Petrou M and Sevilla PG. Image processing: Dealing with texture. John Wiley & Sons, Ltd, 2006.
