# Supplementary material for: Application of Texture Analysis to Study Small Vessel Disease and Blood–Brain Barrier Integrity
Source: Front Neurol. 2017 Jul 19;8:327. doi: 10.3389/fneur.2017.00327 (PMC5515862; doi:10.3389/fneur.2017.00327)
Supplement: Supplementary file 2 [file Data_Sheet_2.DOCX]

**Application of texture analysis to study small vessel disease and blood brain barrier integrity**

**Supplementary Material 2 – Additional results**

**
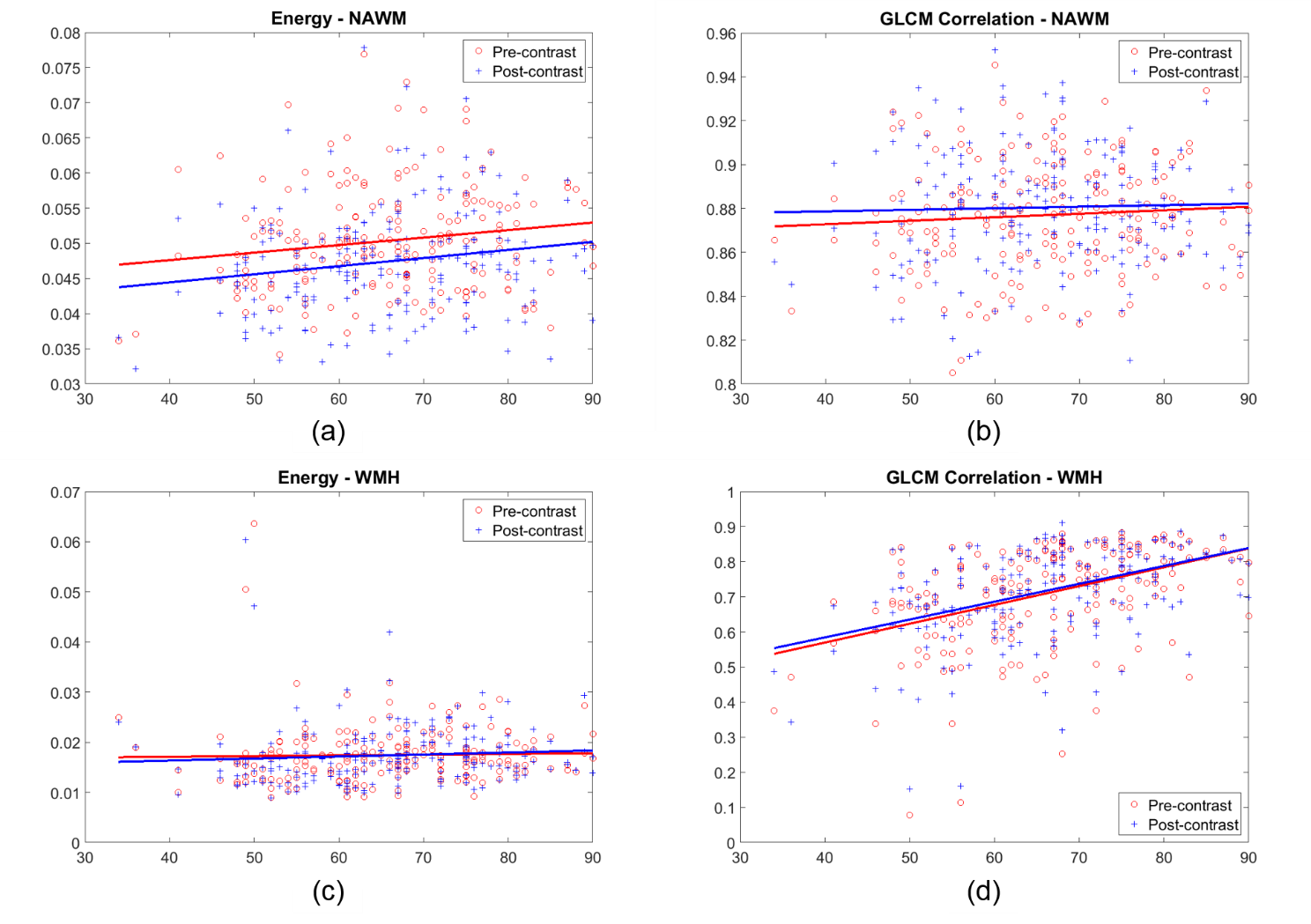
**

**Supplementary Figure S2.1.** Variation of two parameters (out of the 3 selected) that express homogeneity of the texture with age. The graphs show Energy (a and c) and GLCM Correlation (b and d) in normal-appearing white matter (NAWM) and white matter hyperintensities (WMH) obtained from pre- and post- contrast FLAIR images.


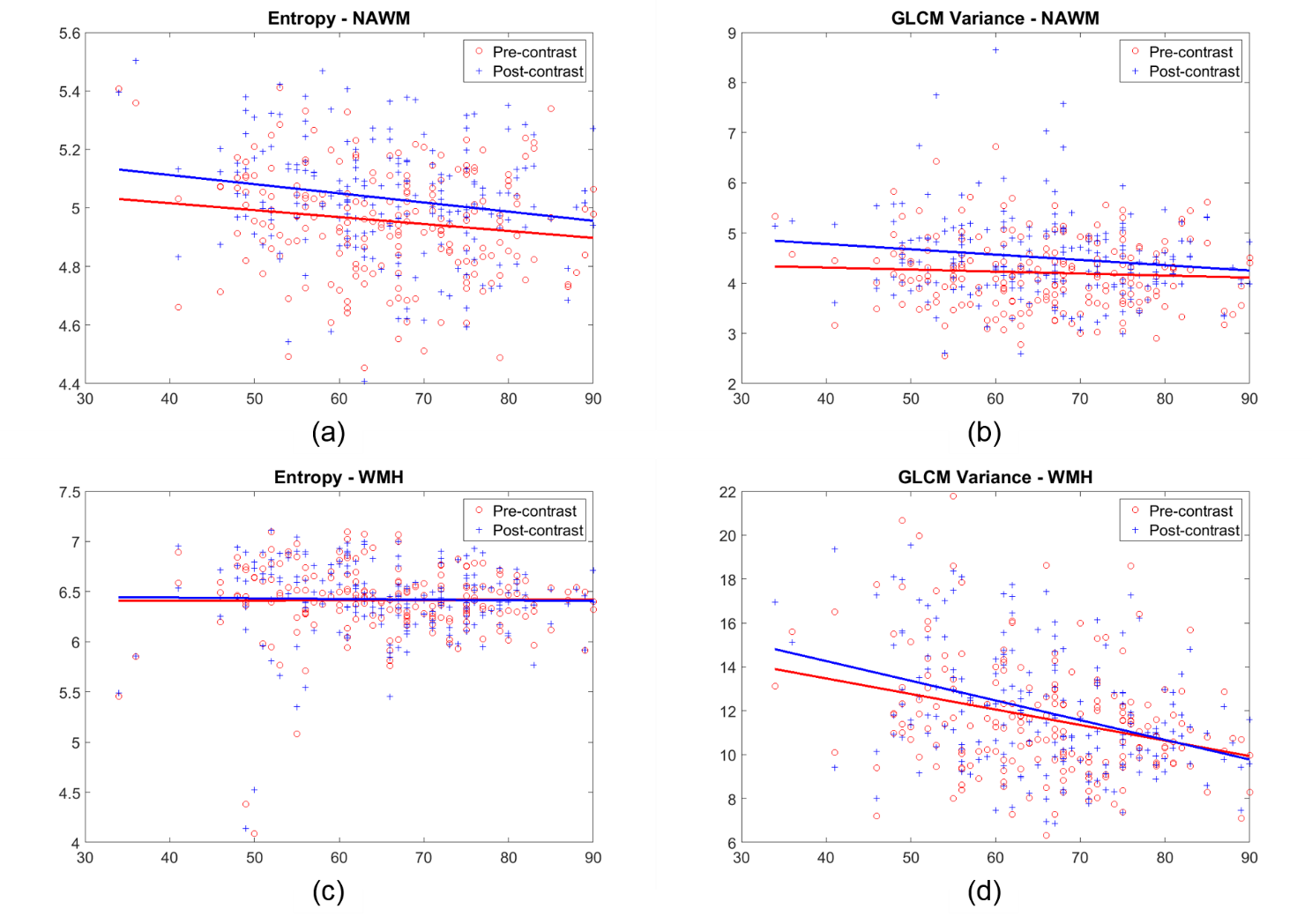


**Supplementary Figure S2.2.** Variation of two parameters (out of the three selected) that express variability of the texture with age. The graphs show Entropy (a and c) and GLCM Variance (b and d) in NAWM and WMH obtained from pre- and post-contrast FLAIR images.

**Supplementary Table S2.1.** Median and interquartile range (expressed as ‘Median; IQR’) of the distributions of all selected texture descriptors

| Texture descriptor | | Pre- / Post- | CSF | Normal tissues | | Abnormal tissues | |
| --- | --- | --- | --- | --- | --- | --- | --- |
|  |  |  |  | NAWM | Deep Grey Matter | Index Stroke lesion | WMH |
| Homogeneity | GLCM correlation | **Pre-** | 0.748; 0.045 | 0.878; 0.032 | 0.886; 0.056 | 0.751; 0.187 | 0.739; 0.177 |
|  |  | **Post-** | 0.760; 0.052 | 0.880; 0.036 | 0.880; 0.048 | 0.764; 0.193 | 0.737; 0.163 |
|  | Homogeneity | **Pre-** | 0.499; 0.047 | 0.713; 0.030 | 0.641; 0.074 | 0.422; 0.169 | 0.492; 0.118 |
|  |  | **Post-** | 0.520; 0.055 | 0.708; 0.037 | 0.635; 0.073 | 0.417; 0.178 | 0.483; 0.121 |
|  | Energy | **Pre-** | 0.017; 0.007 | 0.050; 0.010 | 0.028; 0.010 | 0.015; 0.008 | 0.017; 0.005 |
|  |  | **Post-** | 0.019; 0.007 | 0.047; 0.009 | 0.028; 0.008 | 0.016; 0.010 | 0.016; 0.006 |
| Variability | GLCM contrast | **Pre-** | 5.094; 1.039 | 1.003; 0.175 | 1.885; 0.620 | 7.223; 6.918 | 5.369; 5.331 |
|  |  | **Post-** | 4.332; 1.202 | 1.041; 0.232 | 1.881; 0.561 | 6.880; 6.453 | 5.481; 5.086 |
|  | GLCM variance | **Pre-** | 10.151; 1.290 | 4.142; 0.980 | 7.991; 3.015 | 13.427; 5.237 | 11.287; 3.187 |
|  |  | **Post-** | 9.023; 1.314 | 4.402; 0.959 | 7.955; 2.432 | 13.980; 4.486 | 11.430; 3.609 |
|  | Entropy | **Pre-** | 6.441; 0.332 | 4.960; 0.239 | 5.774; 0.363 | 6.434; 0.544 | 6.448; 0.343 |
|  |  | **Post-** | 6.302; 0.360 | 5.025; 0.230 | 5.797; 0.333 | 6.412; 0.538 | 6.432; 0.398 |

**Supplementary Table S2.2.** Results of the significance (p-values) from the Kruskal-Wallis (K-W) and Median tests for normotensive and hypertensive patients.

|  | | **CSF** | | **NAWM** | | **DGM** | | **Index stroke lesion** | | **WMH** | |
| --- | --- | --- | --- | --- | --- | --- | --- | --- | --- | --- | --- |
|  |  | **K-W** | **Median** | **K-W** | **Median** | **K-W** | **Median** | **K-W** | **Median** | **K-W** | **Median** |
| GLCM correlation | **Pre-Gd** | .962 | .151 | .124 | .749 | **.024*** | **.038*** | .412 | .688 | **.007*** | **.011*** |
|  | **Post-Gd** | .943 | .425 | **.001*** | **.004*** | **.013*** | **.002*** | .455 | .688 | **.001*** | **.004*** |
| Energy | **Pre-Gd** | .359 | .632 | .203 | .151 | **.002*** | **.038*** | .336 | .228 | .281 | .632 |
|  | **Post-Gd** | .635 | .425 | .406 | .338 | **.008*** | **.001*** | .232 | .422 | .115 | .425 |
| GLCM variance | **Pre-Gd** | .990 | .873 | .800 | .749 | .962 | .873 | .558 | .228 | .655 | .494 |
|  | **Post-Gd** | .777 | .873 | .106 | .202 | .469 | .632 | .315 | .688 | .348 | .717 |
| Entropy | **Pre-Gd** | .389 | .632 | .214 | .110 | **.007*** | .079 | .359 | .108 | .551 | .494 |
|  | **Post-Gd** | .549 | .264 | .322 | .202 | **.034*** | **.017*** | .220 | .108 | .218 | .314 |

* p<0.05

**
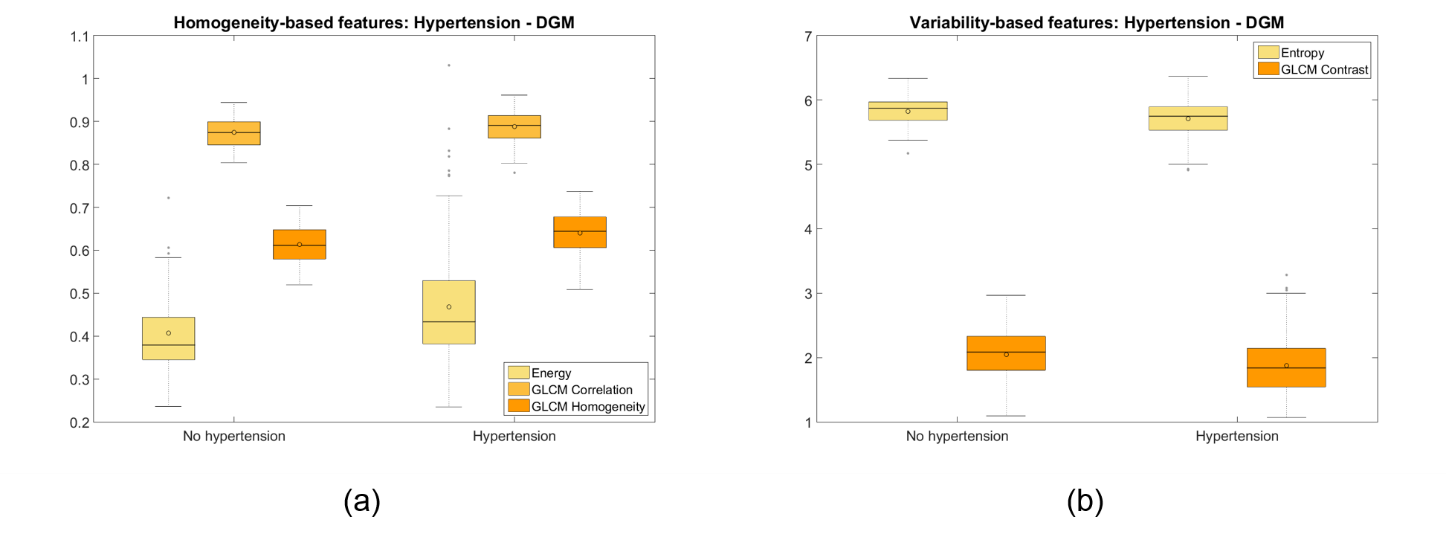
**

**Supplementary Figure S2.3.** Measures of the homogeneity (a) and variability (b) of the texture corresponding to the DGM of hypertensive vs. normotensive patients. Only the Entropy and the GLCM Contrast are shown in (b) because they are the only features that present statistically significant differences. In (a) the Energy has been multiplied by 15 to make it be in a similar range as the GLCM Correlation and GLCM Homogeneity, for visualization purposes

**
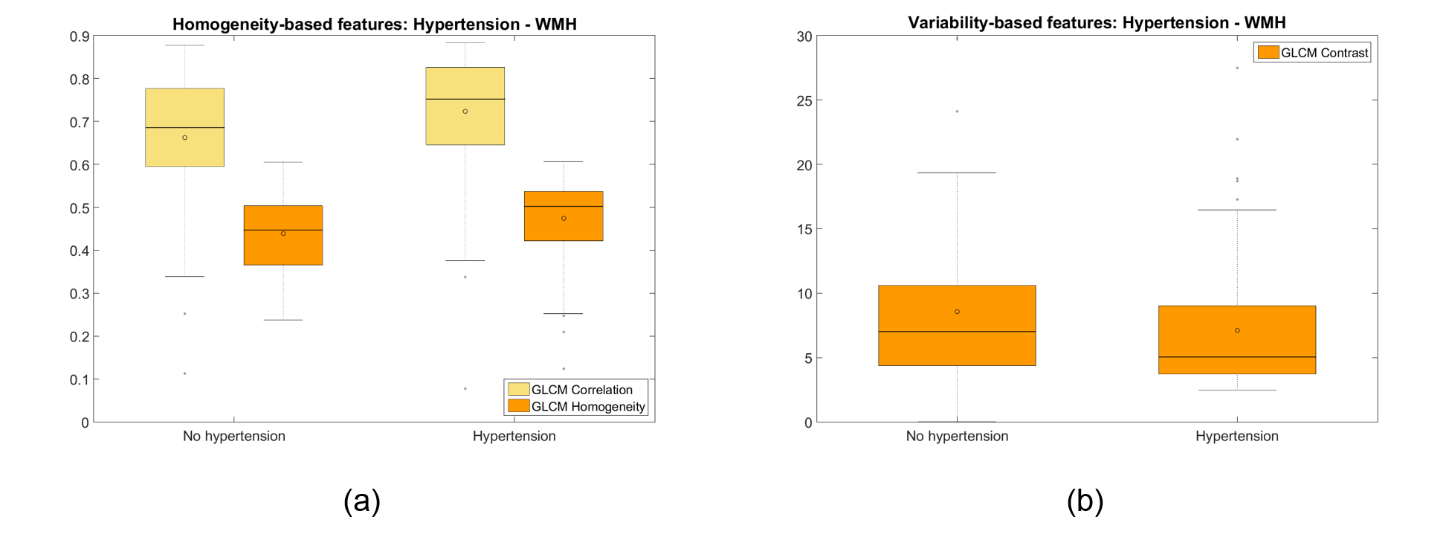
**

**Supplementary Figure S2.4.** Measures of the homogeneity (a) and variability (b) of the texture corresponding to the WMH of hypertensive vs. normotensive patients. Only the GLCM Correlation and the GLCM Homogeneity in (a) and the GLCM Contrast in (b) are shown because they are the only features that present statistically significant differences.

**Supplementary Table S2.3.** Results of the significance (p-values) from the Kruskal-Wallis (K-W) and Median tests for patients that had lacunar stroke vs. those who had cortical strokes.

|  |  | **CSF** | | **NAWM** | | **DGM** | | **Index stroke lesion** | | **WMH** | |
| --- | --- | --- | --- | --- | --- | --- | --- | --- | --- | --- | --- |
|  |  | **K-W** | **Median** | **K-W** | **Median** | **K-W** | **Median** | **K-W** | **Median** | **K-W** | **Median** |
| GLCM correlation | **Pre-Gd** | .822 | .888 | .690 | 1.000 | .186 | .122 | **.001**** | **.001**** | .922 | .939 |
|  | **Post-Gd** | .702 | .673 | .408 | .399 | .482 | .482 | **.001**** | **.001**** | .733 | .720 |
| Energy | **Pre-Gd** | .955 | .673 | .489 | .399 | .546 | .888 | **.001**** | **.001**** | .184 | .325 |
|  | **Post-Gd** | .869 | .673 | .980 | .779 | .295 | .673 | **.001**** | **.001**** | .160 | .206 |
| GLCM variance | **Pre-Gd** | .568 | .888 | .635 | 1.000 | **.048*** | .206 | **.001**** | **.001**** | .241 | .293 |
|  | **Post-Gd** | .938 | .888 | .405 | .261 | **.043*** | .068 | **.001**** | **.001**** | .383 | .183 |
| Entropy | **Pre-Gd** | .800 | .482 | .794 | .779 | .461 | .888 | **.006*** | **.004*** | **.024*** | **.014*** |
|  | **Post-Gd** | .921 | .673 | .844 | .779 | .189 | .206 | **.001*** | **.001**** | **.023*** | **.014*** |

* p<0.05

** p<0.001

**
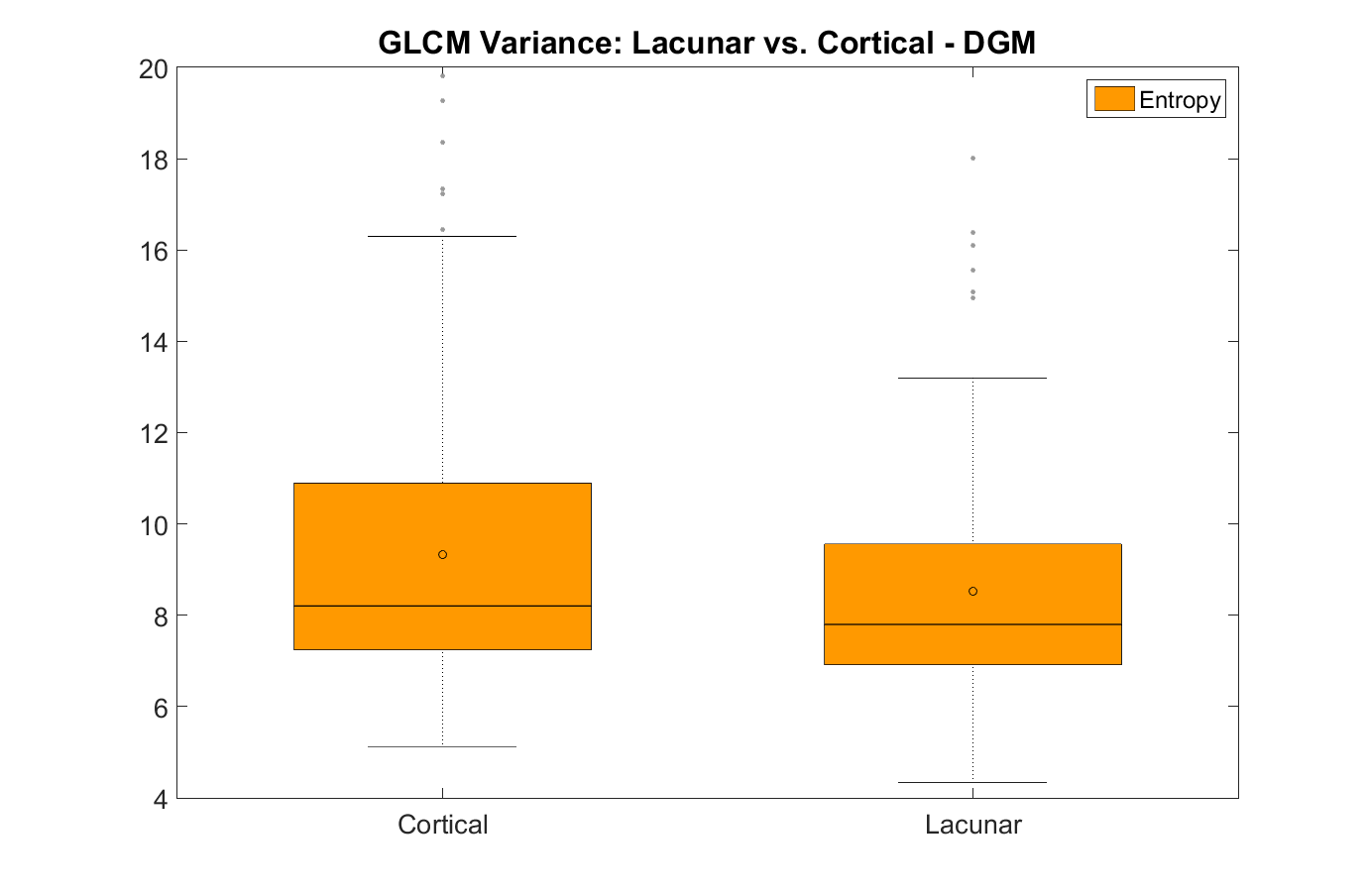
**

**Supplementary Figure S2.5.** GLCM Variance of the texture corresponding to the Deep Grey Matter between patients with cortical or lacunar stroke.

**
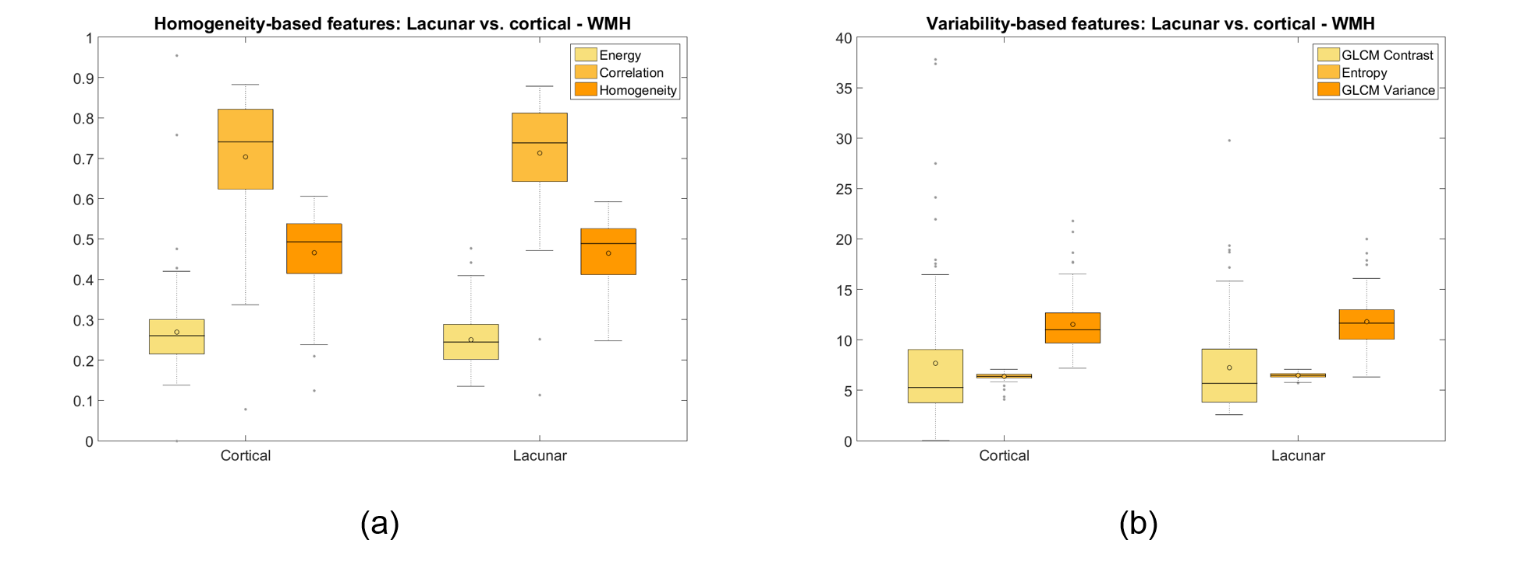
**

**Supplementary Figure S2.6.** Measures of the homogeneity (a) and variability (b) of the texture corresponding to the White Matter Hyperintensities between patients with lacunar or cortical stroke. The energy in (a) has been multiplied by 15 to make it be in a similar range as the GLCM correlation and Homogeneity.

**Supplementary Table S2.4.** Results of the significance (p-values) from the Kruskal-Wallis (K-W) and Median tests for patients with different basal ganglia PVS scores.

|  |  | **CSF** | | **NAWM** | | **DGM** | | **Index stroke lesion** | | **WMH** | |
| --- | --- | --- | --- | --- | --- | --- | --- | --- | --- | --- | --- |
|  |  | **K-W** | **Median** | **K-W** | **Median** | **K-W** | **Median** | **K-W** | **Median** | **K-W** | **Median** |
| GLCM correlation | **Pre-Gd** | .144 | .208 | .078 | .129 | .298 | .149 | .797 | .451 | **.001**** | **.001**** |
|  | **Post-Gd** | .247 | .850 | .078 | .255 | .176 | .271 | .633 | .532 | **.001**** | **.001**** |
| Energy | **Pre-Gd** | **.006*** | .099 | .571 | .897 | **.001*** | **.014*** | **.046*** | .191 | .171 | .261 |
|  | **Post-Gd** | .102 | .202 | .740 | .957 | **.002*** | **.019*** | **.015*** | .168 | .100 | .348 |
| GLCM variance | **Pre-Gd** | **.045*** | .066 | .814 | .434 | .060 | .477 | .140 | .091 | **.001*** | **.004*** |
|  | **Post-Gd** | .268 | .192 | .932 | .707 | **.007*** | .175 | .303 | .663 | **.001**** | **.001*** |
| Entropy | **Pre-Gd** | **.002*** | .075 | .619 | .529 | **.001*** | **.014*** | .059 | .116 | .175 | .700 |
|  | **Post-Gd** | **.045*** | .263 | .583 | .940 | **.001*** | **.023*** | **.019*** | .263 | .142 | .230 |

* p<0.05

** p<0.001

**
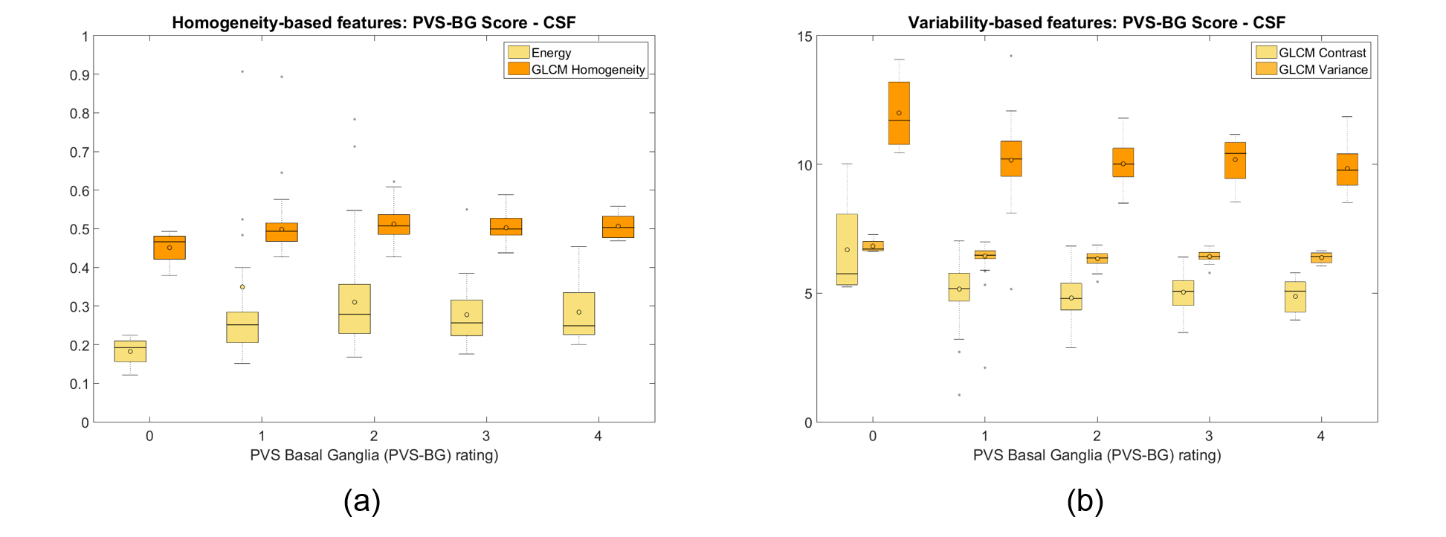
**

**Supplementary Figure S2.7.** Measures of the homogeneity (a) and variability (b) of the texture corresponding to the CSF of patients with different ratings of PVS in the Basal Ganglia. Only the Energy and the GLCM Homogeneity in (a) and the GLCM Contrast and GLCM Variance in (b) are shown because they are the only features that present statistically significant differences. In (a) the Energy has been multiplied by 15 to make it be in a similar range as the GLCM Homogeneity, for visualization purposes.


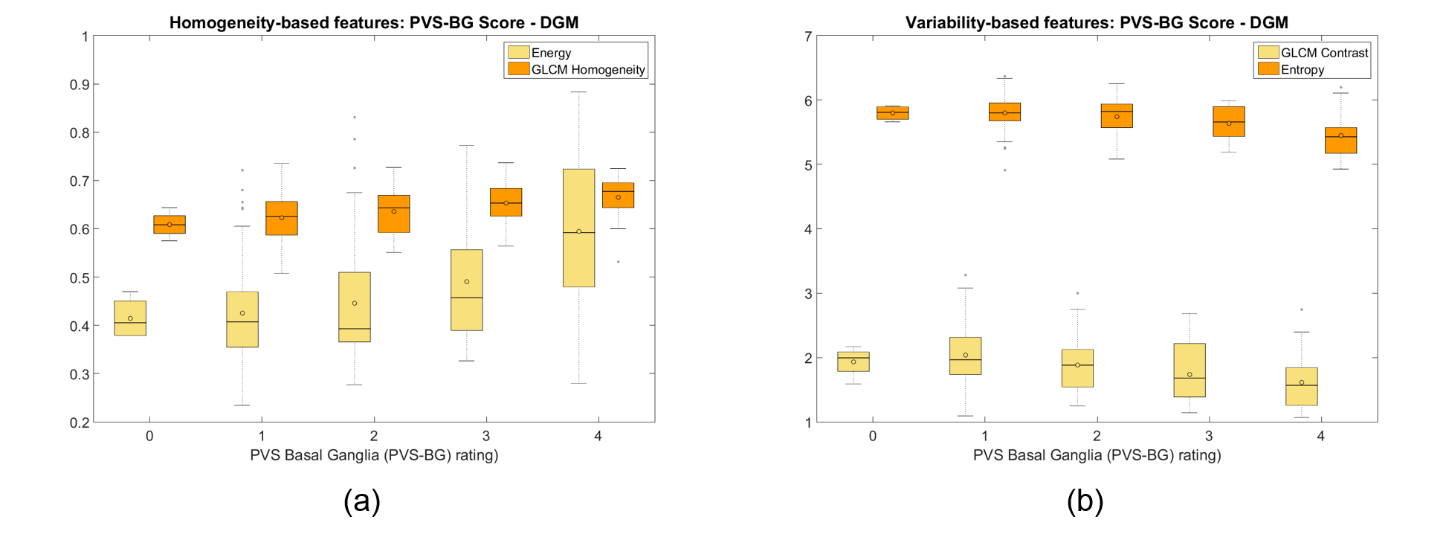


**Supplementary Figure S2.8.** Measures of the homogeneity (a) and variability (b) of the texture corresponding to the DGM of patients with different ratings of PVS in the Basal Ganglia. Only the Energy and the GLCM Homogeneity in (a) and the GLCM Contrast and Entropy in (b) are shown because they are the only features that present statistically significant differences. In (a) the Energy has been multiplied by 15 to make it be in a similar range as the GLCM Homogeneity, for visualization purposes.


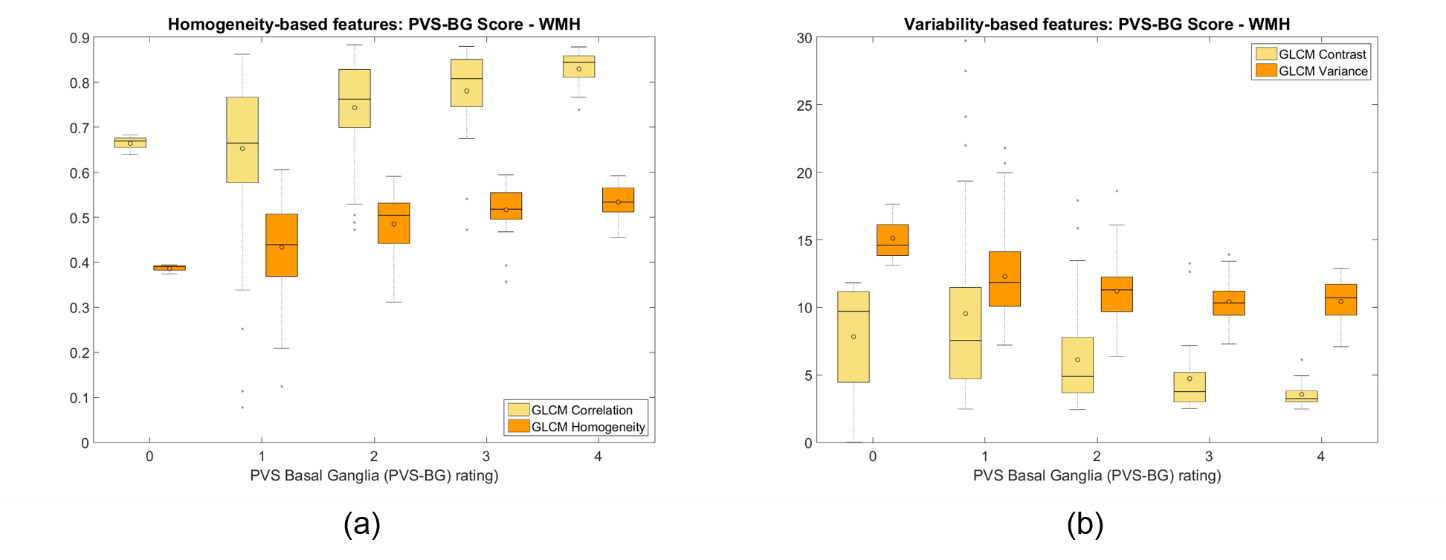


**Supplementary Figure S2.9.** Measures of the homogeneity (a) and variability (b) of the texture corresponding to the WMH of patients with different ratings of PVS in the Basal Ganglia. Only the GLCM Correlation and the GLCM Homogeneity in (a) and the GLCM Contrast and GLCM Variance in (b) are shown because they are the only features that present statistically significant differences.

**Supplementary Table S2.5.** Results of the significance (p-values) from the Kruskal-Wallis (K-W) and Median tests for patients grouped by SVD scores 0 to 4.

|  |  | **CSF** | | **NAWM** | | **DGM** | | **Index stroke lesion** | | **WMH** | |
| --- | --- | --- | --- | --- | --- | --- | --- | --- | --- | --- | --- |
|  |  | **K-W** | **Median** | **K-W** | **Median** | **K-W** | **Median** | **K-W** | **Median** | **K-W** | **Median** |
| GLCM correlation | **Pre-Gd** | **.007*** | .058 | **.015*** | **.023*** | **.004*** | **.009*** | **.029*** | **.021*** | **.001**** | **.001**** |
|  | **Post-Gd** | **.014*** | .196 | **.031*** | .272 | **.002*** | **.005*** | .116 | .081 | **.001**** | **.001**** |
| Energy | **Pre-Gd** | **.003*** | .071 | .555 | .539 | **.003*** | **.024*** | .220 | .187 | .088 | .176 |
|  | **Post-Gd** | **.001*** | **.002*** | .391 | .177 | .064 | .197 | .172 | .388 | .057 | .259 |
| GLCM variance | **Pre-Gd** | .358 | .297 | .405 | .389 | .547 | .800 | .192 | .200 | **.001**** | **.001**** |
|  | **Post-Gd** | **.039*** | **.011*** | .358 | .651 | .081 | .179 | .657 | .693 | **.001**** | **.001**** |
| Entropy | **Pre-Gd** | **.002*** | **.018*** | .441 | .714 | **.001*** | **.008*** | .320 | .272 | .083 | .475 |
|  | **Post-Gd** | **.001**** | **.006*** | .355 | .547 | **.025*** | .121 | .348 | .288 | .104 | .224 |

* p<0.05

** p<0.001

**
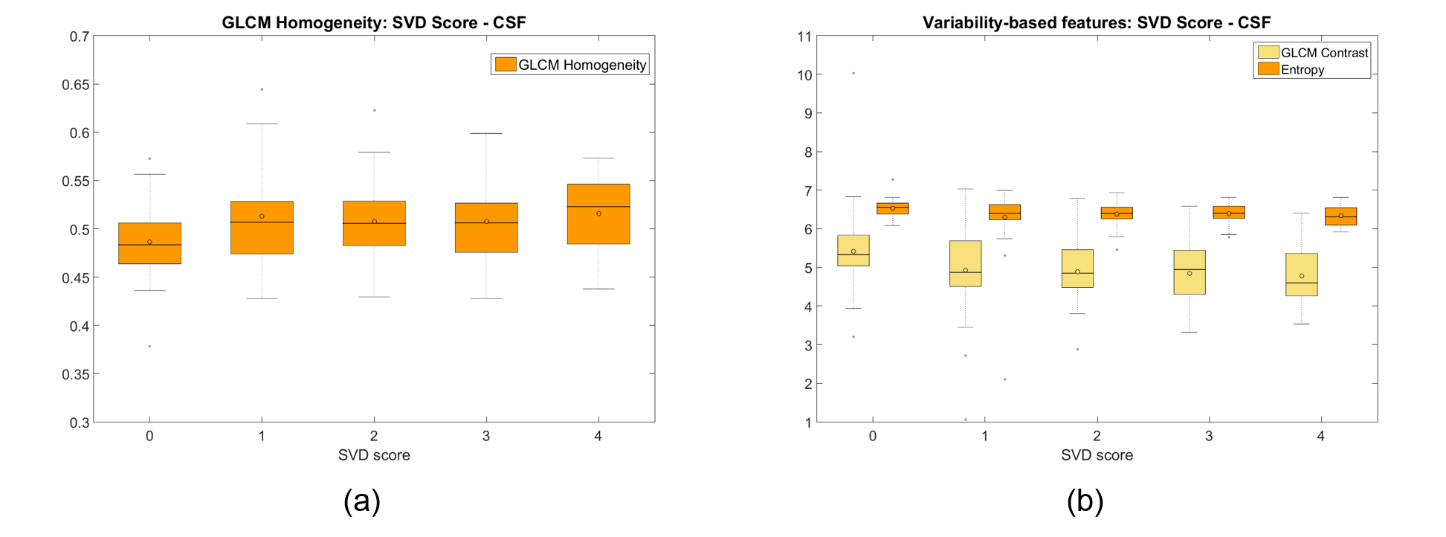
**

**Supplementary Figure S2.10.** Measures of the homogeneity (a) and variability (b) of the texture corresponding to the CSF of patients with different SVD scores. Only the GLCM Homogeneity in (a) and the Entropy and GLCM contrast in (b) are shown because they are the only features that present statistically significant differences.

**
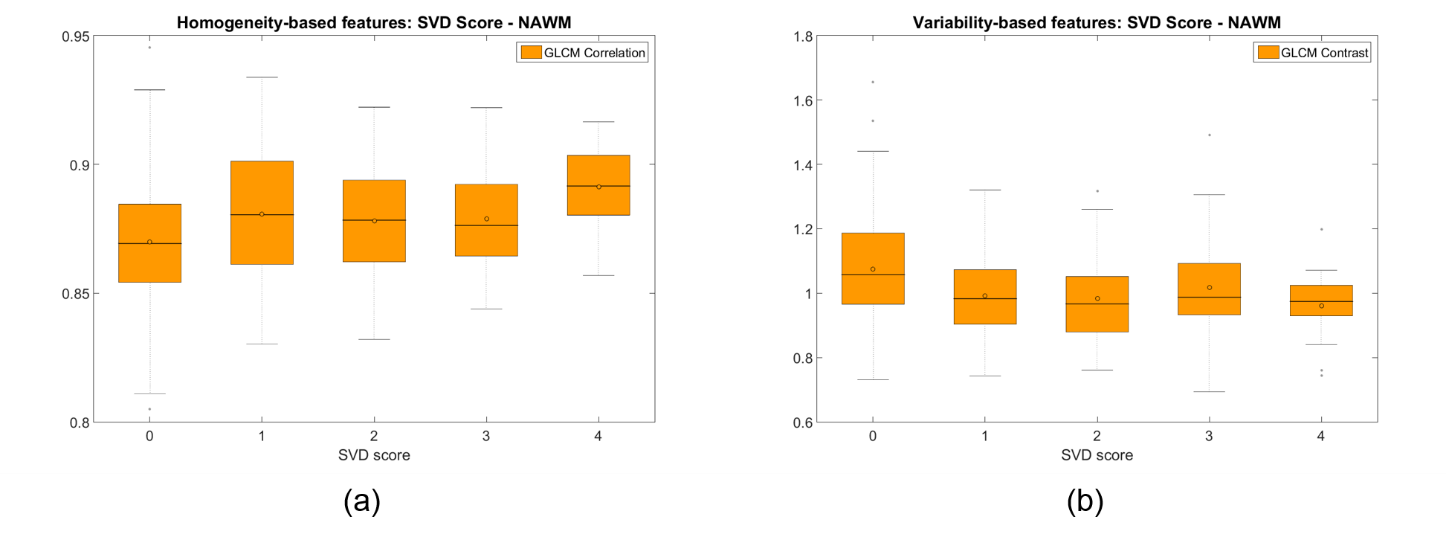
**

**Supplementary Figure S2.11.** Measures of the homogeneity (a) and variability (b) of the texture corresponding to the NAWM of patients with different SVD scores. Only the GLCM Correlation in (a) and the GLCM contrast in (b) are shown because they are the only features that present statistically significant differences.


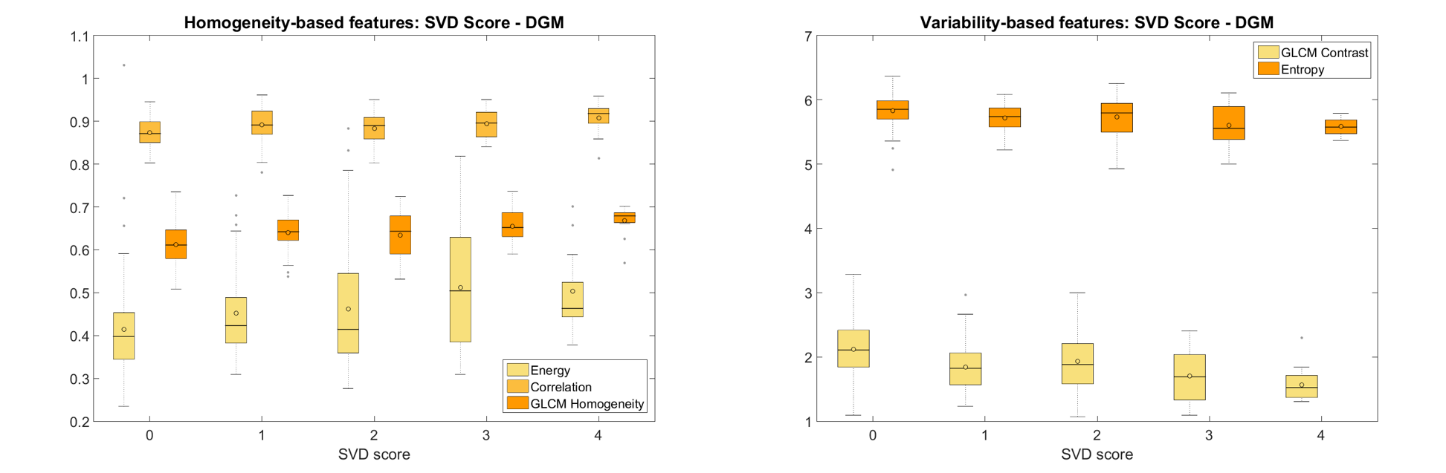


**Supplementary Figure S2.12.** Measures of the homogeneity (a) and variability (b) of the texture corresponding to the Deep Grey Matter (DGM) of patients with different SVD scores. Only the Entropy and the GLCM Contrast are shown in (b) because they are the only features that present statistically significant differences. The values of the Energy have been multiplied by 15 to make it be in a similar range as the GLCM Correlation and Homogeneity, for visualization purposes.

**
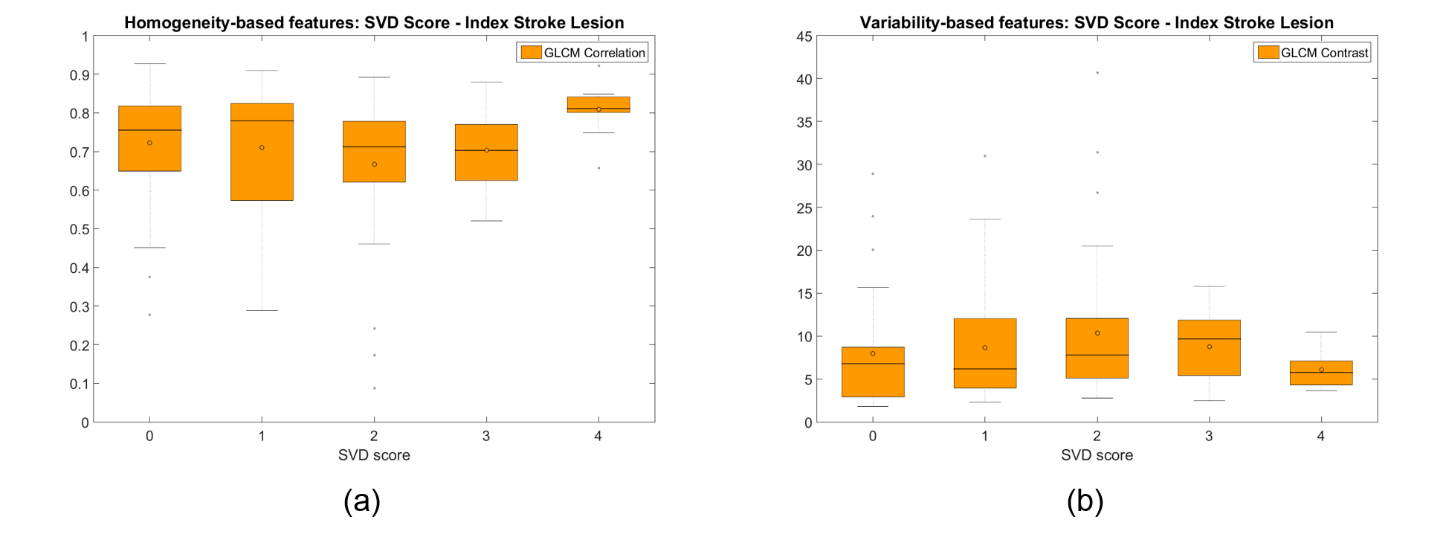
**

**Supplementary Figure S2.13.** Measures of the homogeneity (a) and variability (b) of the texture corresponding to the index stroke lesions of patients with different SVD scores. Only the Entropy and the GLCM Correlation in (a) and the GLCM Contrast in (b) are shown because they are the only features that present statistically significant differences.

**
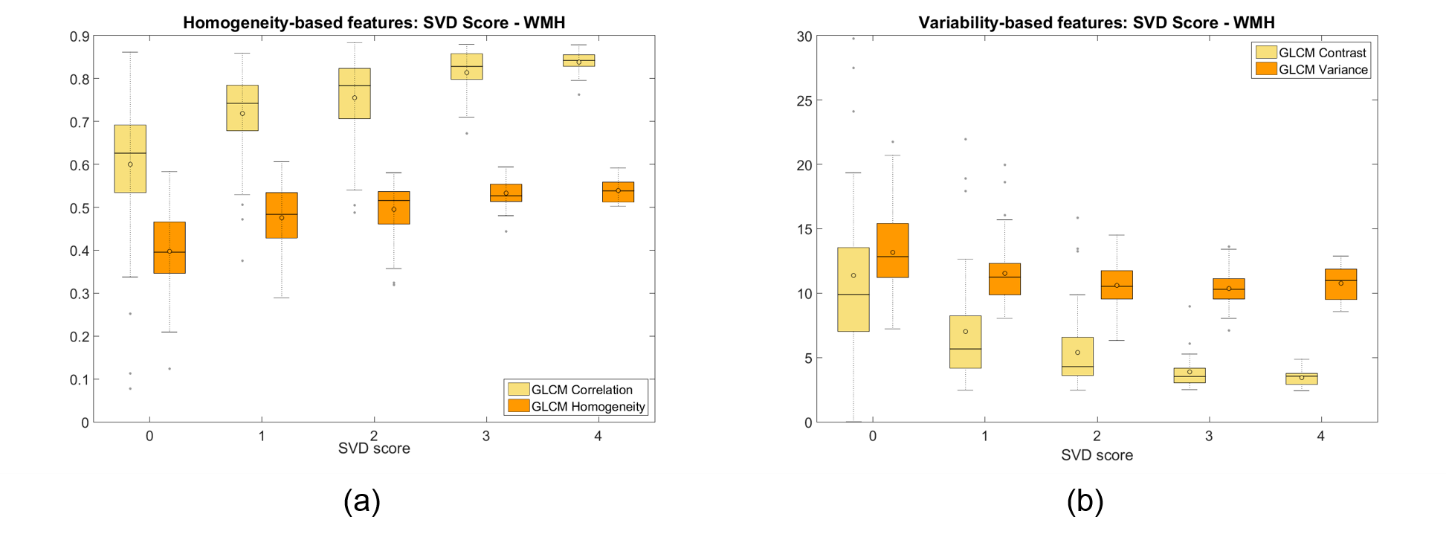
**

**Supplementary Figure S2.14.** Measures of the homogeneity (a) and variability (b) of the texture corresponding to the WMH of patients with different SVD scores. Only the GLCM Correlation and the GLCM Homogeneity in (a) and the GLCM Contrast and GLCM Variance in (b) are shown because they are the only features that present statistically significant differences.

**Supplementary Table S2.6. Summary of the studies where texture analysis has been applied to analyse DCE-MRI.** Results obtained from PubMed and Web of Science until June 2015 using the terms “texture analysis” + “DCE-MRI” after removing duplicates.

| **Ref. no.** | **Sample size and type** | **Age range** | **Pathology studied** | **Contrast agent (type and dose)** | **Scanning sequences (including temporal resolution)** | **Purpose for using texture analysis** | **Texture analysis** | **Other analyses** |
| --- | --- | --- | --- | --- | --- | --- | --- | --- |
| 1 | 41 women (24 with malignant breast tumour and 17 benign) | Not specified | Breast cancer | Gd-DTPA | Sag T1-weighted spgr, 1 series pre-contrast & 3-8 series post-contrast, 90s temp. resol. (minimum); 1.5T or 3T | Tumour type classification (benign or malignant) | 92 **pre-contrast** texture features, 92 **peak contrast** and 92 **kinetic**: gradient (from Gabor, Sobel and Kirsch filters), gray level (1^st^ order) and Haralick (2^nd^ order) features | Support Vector Machine and Prob. Boosting Tree classifiers |
| 2 | 100 women with locally advanced breast cancer, undergoing chemotherapy | 31-77 years (median age: 48 years old) | Breast cancer | Gd-based (0.05 mmol/kg) followed by 20mL saline flush. Injection time: 10 sec. | Sag T1-weighted spgr fat nulled, 10^o^ flip angle, 2 phases pre-contrast and 10 post-contrast, 33.6 sec. (ave) temp. resol.; 3T | Explore tumour responsiveness to chemotherapy | All Haralick features + cluster shade and cluster prominence **on 1-5 min post-contrast data.**  Co-occurring values for 0^o^, 45^o^, 99^o^ and 135^o^ averaged. | Mann-Whitney U and t-tests using the textural features to explore effectiveness separating patient groups |
| 3 | 18 patients (9 men, 9 women) with limb sarcomas | Median age: 54.3 years old | Limb cancer (Leg and arm tumours) | Gd-DTPA (0.1 mmol/kg) followed by N-saline flush | T1-, T2-weighted, DCE-MRI (T1-weighted, 10^o^ flip angle, 100 acquisitions, 2 sec. temp. resol.; 1.5T) | Quantify heterogeneity of tumour enhancement | Coherence (from Haralick) and fractal dimension (from Blanket method) on tumours.  These are **computed from pharmacokinetic and heuristic model-based parametric maps** computed from the DCE-MRI data. | Spearman (correlation between textural features) Wilcoxon test (differences between textural features) and Mann-Whitney U (differences between 2 categories within the same feature) |
| 4 | 234 women (85 with benign lesion and 149 malignant) | 18-78 years (mean age: 46 years old) | Breast cancer | Gd-DTPA (0.1 mmol/kg) followed by 10 ml saline at 3 ml/s | Axial and sag T2-weighted fse, sag T1-weighted non-fat suppressed, DCE-MRI (T1-weighted fspgr fat suppressed, 1 pre-, 9 post-contrast acq., 270 sec. temp. resol.; 1.5T and 3T), DWI pre-contrast | Discriminate malignant from benign breast tumours at 1.5T and 3T | 13 Haralick features on tumours. Texture done in addition to DWI and DCE-MRI from a static sequence **(not known if pre- or post-contrast)** | Diagnostic performance from morphology, kinetic, texture and ADC analyses using SVM, KNN, and random forest classifiers evaluated using ROC curves |
| 5 | 65 women with breast cancer | 31-74 years (mean age: 53.2 years old) | Breast cancer | 2 types of gadolinium-based (0.1 mmol/kg) | 3D T1-weighted fat-suppressed gradient-recalled echo-pulse, every 90-110 sec., 6 acquisitions; 1.5T | Differentiate:  a) Estrogen receptor positive tumours from negative  b) Tumours with viable lymph node metastases after chemotherapy from tumours without nodal metastases | 22 gray level co-occurrence matrix, 11 gray level run length matrix and 76 local binary pattern histogram Fourier features **from both IE (initial enhancement) and PIE (post-initial enhancement) kinetic maps** of the tumours. | Multiparametric feature sets evaluated independently using 6 meta-classifiers: naïve Bayes, decision trees and support vector machine, each using correlation-based and wrapper-based feature subset selection |
| 6 | 121 women (77 malignant and 44 benign lesions) | 21-85 years (mean age: 51.2 years old) | Breast cancer | Gd-DTPA (0.2 mmol/kg), flow rate of 2 ml/s followed by saline flush of 20 ml | 3D T1-weighted spgr, 30^o^ flip angle, non-fat suppressed, one pre-contrast and 5 post-contrast series, 69 sec. temp.resol.; 1.5T | Investigate volumetric (3D) vs. 2D texture analysis approach to characterise breast cancer lesions | All Haralick features for 16, 32, 64 and 128 grey levels in 3D co-occurrence matrices. The texture analysis is performed on **the first post-contrast**  **frame** of the DCE-MRI data | Diagnostic accuracy for each feature determined statistically. Bonferroni correction done afterwards. |
| 7 | 19 patients (9 women, 10 men), 8 with Glioblastoma and 11 with Malignant Glioneuronal tumours | 40-71 years (median age: 57 years old) | Brain cancer | Gd-DOTA (0.1 mmol/kg) | 2D Sag T1-weighted fmpspgr 10^o^ and 90^o^ flip angle pre-contrast and 90^o^ post-contrast during 15 mins. , 28 sec. temp. resol.; 1.5T | Differentiate malignant glioneuronal tumours from glioblastomas | Features from 3 statistical texture analysis methods: gray-level histogram 1^st^ order statistics, Haralick co-occurrence matrix, and run-length distribution matrix, **extracted from post-injection T1w images**. DCE-MRI complementary to texture analysis. | Mann-Whitney U test for group differentiation, principal component analysis and hierarchical ascendant classification done for each class of textural features |
| 8 | 60 women with triple-negative early-stage breast cancer receiving chemotherapy | Mean age: 46 years old | Breast cancer | Gd-DTPA (0.1 mmol/kg) at rate of 2 mL/s | Acquisition at 2 centres:  1)19 post-contrast acquisitions spaced 6-12 mins.  2) Median 5 post-contrast acquisitions spaced 1-5 mins. (Median temp. resol. 2 mins. 46 sec.) | Predict response to chemotherapy in early-stage breast cancer using heterogeneity measures from the grey level co-occurrence matrix obtained from DCE-MRI-derived lesion kinetic maps | 31 features from the grey level co-occurrence matric pre- and post-chemotherapy.  **Three time points were considered: 1) injection, 2) Either 110s or the first post-contrast image (whichever is later) and 3) Last image in series that was no more than 20min after injection.** Texture features were computed from kinetic maps of two classes: (a) empirical parameters (from the three time point rates of wash-in, wash-out and AUC between points 1 and 3; see Figure 2 in the paper) and (b) modelled pharmacokinetic parameters | In addition to textural features, clinical/pathological/ genetic features and semantic morphological features were extracted. Feature selection by logistic regression (Lasso algorithm). Model performance assessed using ROC curves. |
| 9 | 8 women with breast tumours | Not specified | Breast cancer | Gd-chelate (0.2 mL/kg), constant bolus injection during 7 sec. | T1-weighted gradient echo 20^o^ flip angle, 6 acquisitions spaced 120 sec.; 1.5T | Investigate feasibility of using texture analysis to detect malignant tumours | Haralick features determined from pseudoimages generated **from 3 parameters from the 2-compartment model for contrast agent exchange (A, k_ep_ and k_el_)** | Textural parameters input to a feedforward neural network classifier. True positive and negative fractions used to compare gold standard radiological results with results from classifier |
| 10 | 82 biopsy proven (51 malignant, 31 benign) from 74 women | 19 to 82 years old | Breast cancer | 0.2 mmol kg^-1^ gadopentetate  dimeglumine  followed by a 10 ml saline solution flush | Coronal 3D, 1 series before and 5 series post contrast (time interval of 60s). T1W Spoiled Gradient Echo sequence. Repetition time (TR) 8.1 ms, echo time (TE) 4ms; 1.5T | Discriminating malignant from benign lesions | 14 features from GLCM **of three parametric maps: Initial Enhancement, Post-initial enhancement and Signal Enhancement Ratio**. | Lesion enhancement kinetic parametric maps (Initial Enhancement, IE; Post-Initiail Enhancement, PIE and Signal Enhancement Ratio, SER).  Least squares minimum distance classifier |
| 11 | 71 lesions: 43 malignant and 28 benign. Number of women not specified | Not specified | Breast cancer | Not specified (the abstract does not specify anything about DCE) | Not specified | Tumour classification (discriminating benign and malignant) | 8 morphologic parameters and 10 GLCM texture features | Artificial Neural Network. ROC analysis. Feature selection |
| 12 | 23 DCE-MRI parameter maps: 9 low grade gliomas and 14 high grade gliomas | Adults (age not specified) | Glioma | 3ml at 15 ml/sec of Gd-  DTPA-BMA. Dose: 0.1 mmol/kg of body weight | T1w. 3T | One of the experiments was to differentiate low-grade and high-grade gliomas. The other was with simulated data | Fractal dimensions **from DCE-MRI parameter maps** | t-test |
| 13 | 70 clinical cases: 39 probably malignant and biopsied and 31 probably benign and nonbiopsed | Not specified | Breast cancer | 0.1 mmol/kg of Gadopentetate dimeglumine  (Gd-DTPA). | T1w in prone position before and after contrast injection. Five bilateral axial acquisition series were taken at intervals of 111s. 3T. The time points considered for 3TP were 0s, 111s and 444s. | Classification of suspicious breast masses | Multifractal scaling exponent for each clinical  case and log-cumulants reflecting multifractal  information related with texture.  **The first post-contrast images acquired after contrast arrival were used for the multifractal analysis.** | ROC analysis |
| 14 | 58 patients | 35-82 (median=54) | Breast cancer | 0.1 mmol/kg body weight of gadoliniumbased  contrast agent at 2 mL/s, followed by a 20 mL saline flush | T1w with temporal resolution of 1 min acquired on 3T. 1 pre-contrast and 7 post-contrast images with a temporal resolution of 1 min | Predict the clinical and pathological response to neoadjuvant chemotherapy (NAC) in patients with locally  advanced breast cancer (LABC) before NAC is started | 16 GLCM features **at each non-subtracted post-contrast time point** | Kruskal-Wallis test and Mann-whitney U-test. ROC analysis |
| 15 | 81 patients with locally advanced cervical cancer: 49 currently free of disease and 32 relapsed | 32-85 (median=57) | Cervical cancer | Gd-DTPA with a dose of 0.1 mmol/kg body weight followed by saline solution flush | T1w and T2w prior to treatment. DCE-MRI with axial T1w; 1 series pre-contrast and 13 series post-contrast during 5 minutes (first 11 series with sampling interval of 15 sec and the other two with sampling interval of 1 min); 1.5T | Predict if treatment outcome on patients with cervical cancer can be predicted from parameters of the Brix pharmacokinetic model derived DCE-MRI | First-order (21 features). Contrast, correlation, energy and homogeneity from GLCM. Both first-order statistics and GLCM-based features are computed **from each Brix model pharmacokinetic parameter maps (A, k_ep_ and k_el_)** | Pharmacokinetic modelling. Brix parametric maps. Feature selection. Support vector machines. Leave-one-out cross validation. ANOVA and Tukey’s HSD tests |
| 16 | 21 in vivo endorectal MR images from 6 patient datasets. | Not specified | Prostate cancer | 0.1 mmol/kg of body weight of gadopentetate dimeglumine | T2W. The DCE-MRI were acquired during and after contrast agent injection using 3-dimensional Gradient Echo sequence with temporal resolution of 1 min 35 sec; 7 time points; 3T | Segmentation, registration and detection of prostate cancer | Nonlinear dimensionality reduction (using Locally-linear embedding) of pixel intensities, **done at each time point** | Active Shape Model; Affine registration; K-means clustering |
| 17 | 20 rabbits with cholesterol diet and endothelial denudation; 30 extracted segments: 16 contained thrombus (vulnerable) and 14 did not (stable) | N/A | Atherosclerotic plaques; preclinical (rabbits) | Magnevist 0.01 mmol/kg | 2 image modalities of 2D axial T1wBB (T1-weighted black blood) images and DCE-MRI 2D axial before and every 2-3 minutes after injection of contrast agent for additional 7 time points; 3T | Distinguishing vulnerable  versus stable atherosclerotic plaques on DCE-MRI using a rabbit model of atherothrombosis | 352 voxel-wise features: 192 Gabor, 36 Kirsch, 12 Sobel, 52 Haralick and 60 first-order textural features from each voxel over the course of contrast uptake (i.e. **on each time point**) | Minimum-redundancy-maximum-relevance (mRMR) feature selection. Random forest classifier. |
| 18 | 63 benign lesions and 69 malignant lesions in 99 women | From 32 to 85 years old (mean 53.24) | Breast cancer | Gadodiamide, 0.5 mmol/ml or gadopentetate dimeglumine. Flow rate of 4 ml/s at the 5^th^ acquisition | 56 slices each acquisition (total: 35 acquisitions) using a fat suppressed 3-D fast  spoiled gradient echo (FSGR) sequence; 1.5T; | Breast lesion classification (malignant/benign) | 3D shape features and 3D texture features based on the GLCM, on a segmented tumour. **For segmentation, the kinetic and AUC colour maps combined were used** | 3D Shape features (Compactness, margin and ellipsoid fitting); kinetic curve characteristics; binary logistic regression; leave-one-out cross-validation; Kolmogorov-Smirnoff and t-test or Mann-Whitney U test; ROC analysis |
| 19 | 6 women: 4 malignant invasive ductal carcinoma from 4 women, and 4 benign fibrocystic lesion from 2 women | Not specified | Breast cancer | Gd-BOPTA, 0.2 mL/kg bodyweight during 7 seconds. | 64 coronal slices, T1-weighted gradient echo; 1.5T; 6 volumes per dataset were acquired at 6 different time points: The first volume set was acquired  to establish baseline intensity, and another five volume  scans were taken 120 seconds apart | Voxel classification as malignant/non-malignant tissue | 4D co-occurrence –based texture analysis: 14 Haralick features from GLCM with directions (0,0,0,1) (the 4^th^ dimension is the time). Thus, assessing variations in image brightness between the same voxel location **at different times (post-contrast).** | No registration used; Neural Network classifier; ROC analysis |
| 20 | 20 tumours from 18 women: 4 malignant cancer, 6 invasive ductal carcinoma, 10 inflammatory breast cancers | 39-59 years old; mean=48 years | Breast cancer | Gadolinium (0.3 cc/sec at a temporal resolution of 30 s) | Modality not specified. Just used the post-contrast images; acquired at 1.5T. | Breast cancer classification: Pixel-by-pixel classification technique for tumour evaluation. | For each pixel, textures of a block of 8x8 pixels are are characterised based on: Histogram statistics (6 features), GLCM (9 features) and run-length matrices (11 features). 32 intensity bins for GLCM and run-length. Wavelet features: Mean and std from 3 wavelet subbands (low and high pass). **Each textural feature forms a temporal sequence in DCE-MRI** | Segmentation of breast region using active contour model. Feature selection. Support Vector Machines (SVM). ROC analysis |

**References**

[1] Agner SC, Soman S, Libfeld E, McDonald M, Thomas K, Englander S, Rosen MA, Chin D, Nosher J, Madabhushi A. Textural Kinetics: A Novel Dynamic Contrast-Enhanced (DCE)-MRI Feature for Breast lesion Classification. J Digital Imaging 2011;24(3):446-463.

[2] Ahmed A, Gibbs P, Pickles M, Turnbull L. Texture Analysis in Assessment and Prediction of Chemotherapy Response in Breast Cancer. J Magn Reson Imaging 2013;38:89-101.

[3] Alic L, van Vliet M, van Dijke CF, Eggermont AMM, Veenland JF, Niessen WJ. Heterogeneity in DCE-MRI parametric maps: a biomarker for treatment response? Phys med Biol 2011;56:1601-1616.

[4] Cai H, Liu L, Peng Y, Wu Y, Li L. Diagnostic assessment by dynamic contrast-enhanced and diffusion-weighted magnetic resonance in differentiation of breast lesions under different imaging protocols. BMC Cancer 2014;14:366.

[5] Chaudhuri B, Zhou M, Goldgof DB, Hall LO, Gatenby RA, Gillies RJ, Patel BK, Weinfurtner RJ, Drukteinis JS. Heterogeneity in Intratumoral Regions With Rapid Gadolinium Washout Correlates With Estrogen Receptor Status and Nodal Metastasis. J Magn Reson Imaging 2015 [epub ahead of print]

[6] Chen W, Giger M, Li H, Bick U, Newstead GM. Volumetric Texture Analysis of Breast Lesions on Contrast-Enhanced Magnetic Resonance Images. Magn Reson Med 2007;58:562-571.

[7] Eliat PA, Olivié D, Saikali S, Carsin B, Saint-Jalmes H, de Certaines JD. Can Dynamic Contrast-Enhanced Magnetic Resonance Imaging Combined with Texture Analysis Differentiate Malignant Glioneuronal Tumors from Other Glioblastoma? Neurol Res Intl 2012; ID 195176, p.7

[8] Golden DI, Lipson JA, Telli ML, Ford JM, Rubin DL. Dynamic contrast-enhanced MRI-based biomarkers of therapeutic response in triple-negative breast cancer. J Am Med Inform Assoc 2013;20:1059-1066.

[9] Kale MC, Fleig JD, Imal N. Assessment of Feasibilitu to Use Computer-Aided Texture Analysis Based Tool for Parametric Images of Suspicious Lesions in DCE-MR Mammography. Computational and Mathematical Methods in Medicine 2013; ID 872676, p.6

[10] Karahaliou A, Vassiou K, Arikidis NS, Skiadopoulos S, Kanavou T, Costaridou L. Assessing heterogeneity of lesion enhancement kinetics in dynamic contrast-enhanced MRI for breast cancer diagnosis. The British Journal of Radiology 2010; 83:296-306

[11] Ke Nie, Jeon-Hor Chen, Hon J. Yu, Yong Chu, Orhan Nalcioglu, Min-Ying Su. Quantitative Analysis of Lesion Morphology and Texture Features for Diagnostic Prediction in Breast MRI. Academic Radiology 2008; 15(12):1513-1525

[12] Rose CJ, Mills SJ, O'Connor JPB, Buonaccorsi GA, Roberts C, Watson Y, Cheung S, Zhao S, Whitcher B, Jackson A, Parker GJM. Quantifying Spatial Heterogeneity in Dynamic Contrast-Enhanced MRI Parameter Map. Magnetic Resonance in Medicine 2009; 62:488-499

[13] Soares F, Janela F, Pereira M, Seabra J, Freire MM. Classification of Breast Masses on Contrast-Enhanced Magnetic Resonance Images Through Log Detrended Fluctuation Cumulant-Based Multifractal Analysis. Systems Journal, IEEE 2014; 8(3):929-938

[14] Teruel JR, Heldahl MG, Goa PE, Pickles M, Lundgren S, Bathen TF, Gibbs P. Dynamic contrast-enhanced MRI texture analysis for pretreatment prediction of clinical and pathological response to neoadjuvant chemotherapy in patients with locally advanced breast cancer. NMR in Biomedicine 2014; 27(8):887-896

[15] Torheim T, Malinen E, Kvaal K, Lyng H, Indahl UG, Andersen EKF, Futsaether CM. Classification of Dynamic Contrast Enhanced MR Images of Cervical Cancers Using Texture Analysis and Support Vector Machines. IEEE Transactions on Medical Imaging 2014; 33(8):1648-1656

[16] Viswanath S, Bloch BN, Genega E, Rofsky N, Lenkinski R, Chappelow J, Toth R, Madabhushi A. A Comprehensive Segmentation, Registration, and Cancer Detection Scheme on 3 Tesla In Vivo Prostate DCE-MRI. Medical Image Computing and Computer-Assisted Intervention – MICCAI 2008, Lecture Notes in Computer Science 5241:662-669.

[17] Wan T, Madabhushi A, Phinikaridou A, Hamilton JA, Hua N, Pham T, Danagoulian J, Kleiman R, Buckler A. Spatio-temporal texture (SpTeT) for distinguishing vulnerable from stable atherosclerotic plaque on dynamic contrast enhancement (DCE) MRI in a rabbit model. Medical Physics 2014; 41(4):042303-1 - 042303-10

[18] Wang T-C, Huang Y-H, Huang C-S, Chen J-H, Huang G-Y, Chang Y-C, Chang R-F. Computer-aided diagnosis of breast DCE-MRI using pharmacokinetic model and 3-D morphology analysis. Magnetic Resonance Imaging 2014; 32(3):197-205

[19] Woods BJ, Clymer BD, Kurc T, Heverhagen JT, Stevens R, Orsdemir A, Bulan O, Knopp MV. Malignant-lesion segmentation using 4D co-occurrence texture analysis applied to dynamic contrast-enhanced magnetic resonance breast image data. Journal of Magnetic Resonance Imaging 2007; 25(3):495-501

[20] Jianhua Y, Chen J, Chow C. Breast Tumor Analysis in Dynamic Contrast Enhanced MRI Using Texture Features and Wavelet Transform. IEEE Journal of Selected Topics in Signal Processing 2009; 3(1):94-100
